# An Expanded Survey of the Moth PBP/GOBP Clade in *Bombyx mori*: New Insight into Expression and Functional Roles

**DOI:** 10.3389/fphys.2021.712593

**Published:** 2021-10-28

**Authors:** Xia Guo, Ning Xuan, Guoxia Liu, Hongyan Xie, Qinian Lou, Philippe Arnaud, Bernard Offmann, Jean-François Picimbon

**Affiliations:** ^1^Biotechnology Research Center, Shandong Academy of Agricultural Sciences, Jinan, China; ^2^Shandong Silkworm Institute, Shandong Academy of Agricultural Sciences, Yantai, China; ^3^Protein Engineering and Functionality Unit, UMR CNRS 6286, University of Nantes, Nantes, France; ^4^School of Bioengineering, QILU University of Technology, Jinan, China

**Keywords:** insect, Lepidoptera, silkworm, pheromone binding protein, general odorant binding protein, ontogeny, abamectin, vitamin

## Abstract

We studied the expression profile and ontogeny (from the egg stage through the larval stages and pupal stages, to the elderly adult age) of four OBPs from the silkworm moth *Bombyx mori*. We first showed that male responsiveness to female sex pheromone in the silkworm moth *B. mori* does not depend on age variation; whereas the expression of BmorPBP1, BmorPBP2, BmorGOBP1, and BmorGOBP2 varies with age. The expression profile analysis revealed that the studied OBPs are expressed in non-olfactory tissues at different developmental stages. In addition, we tested the effect of insecticide exposure on the expression of the four OBPs studied. Exposure to a toxic macrolide insecticide endectocide molecule (abamectin) led to the modulated expression of all four genes in different tissues. The higher expression of OBPs was detected in metabolic tissues, such as the thorax, gut, and fat body. All these data strongly suggest some alternative functions for these proteins other than olfaction. Finally, we carried out ligand docking studies and reported that PBP1 and GOBP2 have the capacity of binding vitamin K1 and multiple different vitamins.

## Introduction

In insects, the solubilization of pheromone and plant odor molecules before interacting with olfactory receptor neurons (ORNs) is strongly believed to be a *sine qua non* because of the anatomy of the antennal *sensillum* or sensory hair, i.e., the microunit involved in odor detection. In each antennal hair sensillum, an aqueous barrier (sensory lymph) clearly separates each ORN from the pores in the cuticular walls that govern the entry of environmental odor molecules (Picimbon, 2002). The need for the absorption of odor molecules at the surface of the olfactory organ to trap and concentrate the stimuli molecules close to the olfactory receptor (OR) has become the main concept in insect neurobiology, principally in the silkworm moth *Bombyx mori*, where the first sex pheromone (Bombykol) was identified (Butenandt et al., 1959).

Following the discovery of a soluble pheromone-binding protein (PBP) in the antennal sensory lymph of the giant silk moth *Antheraea polyphemus*, it has been postulated that sex pheromone molecules need to interact with PBP in order to activate OR and ORN (Vogt and Riddiford, 1981). The extremely high PBP protein concentration in the sensillum lymph surrounding OR and ORN, pH and pheromone-induced conformational changes in the structure of PBP, PBP-pheromone ligand interaction kinetics and specific mechanisms underlying odor ligand release, resolution of the X-ray crystal structure of *B. mori* PBP1 (BmorPBP1) with the bombykol molecule integrated into the central core of the protein, as well as the notion of supramolecular pheromone-PBP complexes activating OR and PBP-OR co-expression are all in support of a function that is fine-tuned through interaction with sex pheromone molecules and odor chemosensing (Wojtasek and Leal, 1999; Plettner et al., 2000; Sandler et al., 2000; Horst et al., 2001; Lautenschlager et al., 2007; Gong Y. et al., 2009; Krieger et al., 2009). Accordingly, numerous kinetic models with PBP-based sex pheromone deactivation and/or pheromone transport in the moth antennae have been proposed (Vogt et al., 1985; Kaissling, 1998, 2009, 2019; Vogt, 2003, 2005; Gong Y. et al., 2009; Terrado et al., 2019, 2020).

Pheromone-binding proteins and general odorant-binding proteins are very well-recognized members of the larger odorant binding protein gene family, which has been shown to be represented in most insect lineages and species (Picimbon, 2003; Li et al., 2008; He et al., 2011; Iovinella et al., 2011; Donnell, 2014; Ozaki, 2019). Pheromone-binding proteins (PBPs) and general odorant-binding proteins (GOBPs) are particularly notable because (1) they comprise a Lepidoptera-specific clade within the larger insect OBP gene family; (2) they comprise a single gene cluster that arose through early gene duplication; and (3) they are the original genes identified that establish the OBP gene family (Vogt et al., 1989, 1991a,b, 2002; Picimbon, 2003, 2005, 2019; Abraham et al., 2005; Vogt, 2005).

When PBPs and GOBPs were first identified, a major criterion of interest was their antennal specificity: the proteins were localized to the extracellular space of olfactory sensilla and demonstrated to interact with specific pheromone molecules (Vogt et al., 1989; Steinbrecht et al., 1995; Plettner et al., 2000; Zhang et al., 2001; Nardi et al., 2003; Wang et al., 2003; Zhou et al., 2009). The noctuid *PBP/GOBP* clade was maintained despite sex pheromone divergence, speciation, and species recognition (Picimbon and Gadenne, 2002; Picimbon, 2003; Abraham et al., 2005; Allison and Cardé, 2016). It was, however, the antennal specificity that argued strongly that the proteins were involved in olfactory functions and, therefore, had some major role entirely strictly tuned to the detection of odor molecules. Originally, PBP and GOBP in adult moths were considered to be absent from the thorax, midgut, fat body, and metabolic tissues but abundant in the antennae and legs; this also included proteins/genes of the current study, namely, “PBP1, PBP2, GOBP1, and GOBP2” of the silkworm moth, *B. mori* (BmorPBP1, BmorPBP2, BmorGOBP1, and BmorGOBP2; Vogt et al., 1991a,b; Krieger et al., 1996; Sandler et al., 2000; Forstner et al., 2006; Gong D. et al., 2009; Zhou et al., 2009; Li et al., 2012; Xuan et al., 2014; Picimbon, 2019). In addition, while the larval expression of GOBP2 was restricted to large sensilla basiconica, sensilla styloconica, or other gustatory chemosensilla from maxillary palps and antenna (Vogt et al., 2002), the adult expression of BmorGOBP2 was shown to be associated with the female moth pheromone gland (Xuan et al., 2014), strongly suggesting multiple functions for this protein. Gel digestion of SDS-PAGE-separated proteins and liquid chromatography coupled to tandem mass spectrometry (Nano-LC/MS/MS) analysis showed the presence of GOBP2 with many other OBPs (OBP6, OBP56d, PBP-related protein 3, sericotropin, and protein B1) in a library of more than 9,000 peptides from the *Bombyx* pheromone gland (Xuan et al., 2014), urging us to use molecular biology and quantitative real-time PCR as an alternative/complementary approach of biochemistry and immunoblotting to focus on the PBP/GOBP clade. The occurrence of GOBP2s and other OBPs not only in insects but also in the kingdom of bacteria emphasizes their involvement in various additional non-olfactory tasks (Liu and Picimbon, 2017; Picimbon, 2019). Moth PBPs and GOBPs display about 30–79% identity with “pheromone/odorant binding proteins” from *Acinetobacter baumannii* and *Macrococcus caseolyticus* (OIE61716, RKO12089, RKO12629, RKO12557, RKO12708, RKO12709, WP_170831700; Genetic locus: LYIE01000111, RBVL01000056, RBVL01000061, RBVL01000065, and RBVL01000098). Therefore, it could be that non-olfactory function is a very general feature of the OBP protein gene family. Many members of the OBP family have since been shown to be expressed not only in the antennae and legs but also in many various metabolic organs, including the thorax, midgut, and fat body (Li et al., 2008; Ribeiro et al., 2014; Sun et al., 2018; Rihani et al., 2021).

We report further on these studies in relation to multifunction and OBP genes. First, we assay the sex pheromone responsiveness to make an inference based on temporal analyses of PBP/GOBP expression. Then, we report on our entire study in relation to the temporal and tissue-specific expression of four OBPs, two pheromone-binding proteins (PBPs), PBP1 and PBP2, and two general odorant-binding proteins (GOBPs), GOBP1 and GOBP2, of the domesticated silkworm moth *B. mori*. Contrary to the strong widespread belief that these proteins in the moth PBP/GOBP clade are expressed exclusively in adult (predominantly male) antennae and used exclusively for pheromone binding and sex recognition, we show data that the respective genes, and in many cases also the proteins, are expressed in multiple larval, pharate adult, and adult tissues as well as in non-sensory tissues of young adult males subjected to immersion into a specific anthelminthic/insecticide (abamectin, Avermectin B1) solution. This “abamectin” experiment is crucial to cover the gene expression of PBPs and GOBPs in the thorax, gut, and fat body as well as some age- and chemical stress-dependent conditions. Due to such unusual findings, we also carry out ligand docking studies and report that BmorPBP1 and BmorGOBP2 have the capacity to bind vitamin K1 and multiple different vitamins, respectively.

## Materials and Methods

### *Bombyx mori* Rearing and Tissue Dissection

Original collections of silkworms were made from Qingsong × Haoyue crossbreeds (Yantai, Zhifu, Shandong Province) maintained from the egg stage and throughout larval development in growth chambers at 23°C with 70% relative humidity and a photoperiod of 15-h light: 9-h dark. The larvae were reared on a layer of mulberry leaves until spinning prior to being sexed and transferred to laboratory conditions. Fifth-instar larvae were used for tissue collection. In the laboratory (Jinan, Shandong Sheng), male and female “cocoons” were maintained at room temperature. The pupae were kept separately in two batches (males and females) in two different rooms, both held at 25°C and 15 h light: 9 h dark. Female pupae were used for tissue extraction (antennae, fat body, gut, head, legs, pheromone gland, epidermis, cuticle, thorax, and wings) at five different stages of the adult development, e.g., 1- to 5-days before emergence, E-1 to E-5, following Vogt et al. (1993); Dedos and Fugo (2001) and Picimbon et al. (2001). For the antennae and legs, cuticle deposition, which was initiated in the early stages, was observed under a microscope as a change in the external structure. On E-5, the moth tissues had a soft yellowish-white appearance/coloration and no cutinized layer (Picimbon et al., 2001). On E-4 (abdominal cuticle deposition), E-3 (eye pigmentation), and E-2 (formation of legs), the coloration and rigidity of the nymph body changed to nearly reach the adult form (imago stage: E-1, antenna, and wing pigmentation).

At the adult stage, the males and females were also kept separately. When the adults eclosed, no newly born (D0) male and female were paired. Therefore, all data related to unmated status. Males and females were dissected for antennae, head, legs, wings, thorax, the abdomen, and, in females, the pheromone gland at precise age after emergence. Epidermis, gut, and fat body tissues were extirpated from the abdomen. The anterior, median, and posterior legs were also collected separately. All the legs were cut off at the femur-tibia (tarsal segments) articulation. Compound eyes were removed from the cephalic capsule. We also collected eggs laid by 8-day-old virgin females and unlaid eggs in the female abdomen (ovarian tissue) that were kept separately. Hemolymph and meconium were aspirated with a micropipette after pressure on the abdomen and diluted in water. The cocoons were cut into small pieces and heated in a boiling water bath for several hours before collecting protein samples. Antennae were harvested at the same time each day during the hours of light (Ichikawa, 1998; Ichikawa and Ito, 1999). All the organs and tissues were frozen and stored at −80°C until protein or RNA extraction.

In a mixed cocoon population, both silkworm adult males and females became active even under light conditions as soon as they have emerged from the cocoons. The females stayed nearby the cocoons, rising wings, and expelling ovipositor and pheromone gland that, soon enough, will draw the males to their vicinity. Newly emerged males fanned their wings and walked immediately to *Bombyx* females ready for mating. The expected adult lifespan of the Qingson x Haoyue strain is ~10–15-days, with mating activities significantly decreasing after 7–9-days. Unmated females lay eggs after 7–9-days as described by Osanai (1978).

### Olfactometer Behavioral Studies

In the pheromone response tests, adult males of different ages (1- to 8-day-old) were placed individually in an I-shaped tube olfactometer connected to a separated glass chamber housing one young (1-day-old) virgin female in a calling posture. The same female was used to test a series of 10 males. Thirty-forty males were tested for each category. We tested 9-day-old males, we noted pheromone responsiveness but we did not consider the result for statistical analysis (sample size *n* = 10). Old males d16–18 were also assayed but exhibited no activity (*n* = 10). The characteristics of the female adopting a calling posture were wing vibrations, intense pulses of abdominal movements, and pheromone gland extrusions at the abdominal tip (Ichikawa, 1998). The source chamber was covered to exclude visual stimuli. In the open I-track olfactometer tube (20-cm long with a diameter of 3 cm), humidified and pre-cleaned air was constantly blown at a total flow of 2.5 l/min (air pump vacuum cleaner AG 1605; Beijing-Keep Science Analysis & Technology, Co. Ltd.). Tests were conducted at room temperature (25°C) during the light period (photophase) of the silkworms. The females also sustained calling behavior and pheromone production during photophase (Ichikawa and Ito, 1999). The male was introduced in the upper part of the olfactometer once the female flapped wings, lifted abdomens, and expelled the sex pheromone gland every several seconds. Male behavioral responses were evaluated using two criteria: (1) time reaction (Tr: the male left the upper part of the tube, crossing the arbitrary point in the reaction zone, 18 cm away from the odor source) and (2) time to reach the female odor source (Ts: the male reached the other extremity of the tube, touching the zone connected to the calling female). The behavior score test lasted for 2 min. The data were statistically analyzed using a Mann-Whitney U test at *p* < 0.001.

### Quantitative Real-Time PCR (qRT-PCR)

For the measurement of gene expression in adult tissues, complementary DNAs were synthesized from antennal RNA (1 μg) using M-MuLV transcriptase (Fermentas, Waltham, MA, United States). The qRT-PCR was carried out using a StepOnePlus ABI7500 (Thermo Fisher Scientific, Waltham, MA, United States) system. The thermocycler program had an initial denaturing step of 2 min at 95°C followed by 40 cycles of 5 at 95, 20 at 60, 30 at 72, and 15 s at 95°C. A melting curve analysis was performed by monitoring fluorescence (SYBR Green I; Takara Bio Inc., Shiga, Japan) at 60°C for 1 min as suggested by the instructions of the manufacturer. Using 60 insects per age, three mRNA samples were collected (yielding three separate cDNA samples). Each sample taken for RNA extraction and cDNA synthesis corresponded to 20 moths equivalents.

Each of the three resulting reaction samples was run in triplicates. Specific primers were designed to yield amplicons of about 130–200 bps: BmorPBP1 (#X94987) sense 5′-tttgccaagaaacatggagc-3′, antisense 5′-tgtggatttcagctttgaagc-3′; BmorPBP2 (#AM403100) sense 5′-ggaaaagctcacgagtttgc-3′, antisense 5′-gaccttcagtgttctttcgca-3′. The BmorGOBP1 (#X94988) and BmorGOBP2 (#X94989) primers were same as those used for one-step RT-PCR. Controls used cyclophilin A and actin primers described in *One-Step Reverse transcriptase PCR (RT-PCR)*. Primers to additional sensory and non-sensory genes, such as *antennal oxydase-1* (AOX1), *antennal esterase-1* (AE41), *JH esterase* (JHE), *cytosolic juvenile hormone-binding protein* (cJHBP), *hemolymph JHBP* (hJHBP), *ecdysone receptor variant B1* (EcR-B1), *pheromone olfactory receptor-1* (OR1), and *cytochrome P450* (CYP306A1 and CYP4M9) were as follows: BmorAOX1 (NM_001110342, 5′-gatctgaccgtattcaaagc-3′, 5′-gcaaagtcttcttccacgtt-3′), BmorAE41 (NM_001130880, 5′-tttggccgtttgaaatcagc-3′, 5′-gcttgctttccatgttggaa-3′), BmorJHE (AF287267, 5′-tccataatggaggtgaaagc-3′, 5′-tgctcatggacgtcagtaat-3′), BmorcJHBP (NM_001044203, 5′-gtctgaagtatgttgaggct-3′, 5′-aaagtcagtagaccgttcca-3′), BmorhJHBP (NM_00143609, 5′-actaaagcgaagacggtgc-3′, 5′-tgtagccatacctgacagc-3′), BmorEcR-B1 (NM_001173375, 5′-aggtatctttcggagaagct-3′, 5′-ccaagtctgcgttactcttt-3′), BmorOR1 (NM_001043410, 5′-tcgcttcataacggtaatgc-3′, 5′-ccataaggatccgaaaatgc-3′), BmorCYP306A1 (NM_00111275, 5′-aaatacaggaggaaggatgc-3′, 5′-ccacggactagaacttcaat-3′), and BmorCYP4M9 (NM_001079666, 5′-aatgggccgtattttaagc-3′, 5′-ggtcaaacacaagaggatct-3′).

All the qRT-PCR products were sequenced to attest to the specificity of the amplicon. Gene expression levels were calculated relative to the *actin* gene using the 2[–ΔΔC(T)] method and following Livak and Schmittgen (2001) and Xuan et al. (2015). In using the 2[–ΔΔC(T)] method, it was mandatory to use a single set of primers and compare specific gene expression with actin across different age or tissue samples (Step 1). The final quantitative real time-PCR data were statistically analyzed by one-way ANOVA with the SPSS software. In Step 2, we compared the average value of the specific gene expression to that of PBP1 (calibrated to 1) across different ages and antennal samples to see or monitor the peak of PBP or GOBP expression in the same experiment (see Supplementary Figure 1). For comparison of tissues after insecticide exposure, we analyzed each gene separately. Expression in the antennae was calibrated to 1 (Step 2, **Figure 5C**) to see or monitor the peak of PBP/GOBP expression in a specific tissue after chemical stress.

### One-Step Reverse Transcriptase PCR (RT-PCR)

For the measurement of gene expression in the egg, larva, and pupa tissue samples, total RNAs from all the various tissues were isolated using the Trizol^TM^ method (Invitrogen, Waltham, MA, United States). RNA quality was assessed by optical density measurements (Eppendorf BioPhotometer; Eppendorf, Hamburg, Germany) and electrophoresis on agarose gel (1 μg). The total RNAs were then used as templates in specific one-step-reverse transcription PCR experiments (Takara Bio Inc., Shiga, Japan). For the samples taken for RNA extraction and one-step RT-PCR, three 1.5-ml Eppis tubes full of eggs and about 50 larvae and fifty pupae (per pre-eclosion stage) were required.

The RT-PCRs were performed on total RNA sample (100 ng) in a TaKaRa PCR Thermal Cycler Dice (Takara Bio Inc., Shiga, Japan) under optimal conditions: reaction cycles at 50°C for 30 min, 94°C for 2 min, 40 cycles of 94°C for 30 s, 60°C for 30 s, and 72°C for 40 s. Test primers were: BmorPBP1 (#X94987) sense 5′-gagatgacgctaacagatgc-3′, antisense 5′-ttcagctttgaagcaggtcg-3′; BmorPBP2 (#AM403100) sense: 5′-gcaatcctgtgcatgtccaa-3′, antisense 5′-agacctctgccattaagagc-3′; BmorGOBP1 (#X94988) sense 5′-caagttcgaacacagagagc-3′, antisense 5′-gcgtccttgaaacattcagc-3′; BmorGOBP2 (#X94989) sense 5′-taagacccttgaggaatgcc-3′, antisense 5′-ttttctcagctagaacttgcc-3′. *Cyclophilin A* and *actin3* genes from *B. mori* were both amplified alongside the test genes to calibrate for both experimental variability and RNA integrity. Control CypA and Actin primers were: CypA (#NM_001043836) sense 5′-cgagaatttcacccttaagc-3′, antisense 5′-catgccttcaacaacattcc-3; Actin (#X04507) sense 5′-gacatggagaagatttggc-3′, antisense 5′-agtcattcgtcagataacgg-3′. The one-step RT-PCR products were analyzed on 1% agarose gel, visualized using ethidium bromide staining, gel-purified (TIANgel Midi purification kit; Tiangen, Sichuan, China), and cloned into a pGEM-T Easy vector (TransGen Biotech, Beijing, China) before they were sequenced on an ABI3700 sequencer instrument using an RR Dye Deoxy terminator cycle sequencing kit (PerkinElmer, Waltham, MA, United States) and specific primers.

### Protein Analysis

Biochemical studies were preliminarily conducted on fractions of soluble proteins extracted from eggs, gut, head, mouthparts, epidermis, silk gland, and tail-end spine, as well as thoracic and abdominal legs from fifth instar silkworm larvae. There were not enough proteins to perform SDS-PAGE and immunoblotting, even in concentrated samples. Subsequently, highly concentrated protein samples were used from calling virgin 4-day-old female adult tissues (fat body, eggs, gut, head without antennae, legs, epidermis, thorax, and wings). In further experiments, anterior, median, and posterior legs were dissected from a pool of fifty 5-, 6-, 7-, 8-, and 9-day-old unmated females, providing 1 mg/ml of various age-dependent leg protein samples. Proteins were also extracted from the anterior, median, and posterior legs of fifty 8-day-old males from another pool of silkworms. In this pool of silkworms, the tarsi and femur/tibia of males were dissected at the same time as those of females (8-day-old). From these insects, the antennae, head, eyes, cephalic capsule, sex pheromone gland, hemolymph, and meconium were also collected.

In the preparation of protein samples, tissues were freeze-dried in liquid nitrogen and crushed to powder with mortar and pestle in a specific protein extraction buffer (20 mM Tris-HCl, pH 7.4, containing 100 mM of phenylmethylsulfonyl fluoride, PMSF). The tissue homogenates were centrifuged (Neofuge 15R; HealForce, Shanghai, China) at 12,000 g for 10 min at 4°C to collect the protein supernatant. The protein concentration in the supernatant was measured by Bradford assay. Using larval tissues, the following protein concentrations (in μg/μl) were determined: anterior legs (2.99), median legs (5.24), posterior legs (3.95), gut (3.4), head (2.71), mouth (3.55), the epidermis (5.07), silk gland (0.49), and tail (1.37). Tissue-specific ~16 kDa protein bands were observed, but no labeling was found in the first attempts to immunoblot. The relevance of this was linked to the approximate molecular weight of PBPs/GOBPs, i.e., 15.89–17.17 kDa (without the signal peptide).

Using adult tissues, the following protein concentrations (in μg/μl) were determined: wings (1.08), legs (1.95), head (1.46), thorax (5.25), the abdominal epidermis (6.83), fat body including eggs (14.66), and gut (3.03). The protein solution was then concentrated by lyophilization (Labconco, Kansas City, MO, United States). After freeze-drying, the protein powder corresponding to 1-, 5-, 10-, 20- and 40-fold concentrated samples were resuspended in 20 μl of a 5x SDS (denaturing)-loading sample buffer, boiled, and loaded onto a 15% acrylamide gel under denaturing conditions. SDS-PAGE was run at 120 V for 2.5 h. All tissue samples that allowed for visualization of a protein band in the zone corresponding to the 14–24 kDa markers (14–100 kDa Blue Plus^®^ II Protein Marker; TransGen Biotech Company, Beijing, China) were selected for immunoblotting.

Immunoblotting was performed to check for the detection of BmorPBP1, BmorGOBP1, and BmorGOBP2 in concentrated protein samples of various tissues. No antibody was available for the detection of BmorPBP2. Accordingly, four aliquots per tissue were prepared for protein analysis and immunodetection. Polyclonal antibodies against these proteins were from Maida et al. (2005). Sodium dodecyl sulfate-polyacrylamide gel electrophoresis SDS-PAGE and Western blotting were performed with traditional biochemical methods. After the SDS-PAGE, proteins were transferred to pure nitrocellulose blotting membranes (Pall Corporation, Port Washington, NY, United States) using a system from Beijing Junyi-Dongfang Electrophoresis Equipment Co. Ltd. (Beijing, China), as described in Xuan et al. (2014). Protein was detected using an HRP-DAB chromogenic substrate detection system (Tiangen, Sichuan, China) as described by the protocol of the manufacturer. Blocking was done in TBST (10 mM Tris-HCl, 0.15 M NaCl, 0.05% Tween-20) overnight at 4°C. Primary and secondary antibody sera were used at dilutions of 1:2,000 and 1:10,000, respectively. Unbound antibodies were washed off, leaving only signals corresponding to antibodies bound to the protein. The specificity of antibody cross-reactivity with electrophoresed bands was confirmed by the position of molecular mass markers (visualization of both the 14–24 kDa marker and sample proteins on the same gel or Western blot; prestained Blue Plus^®^ II Protein Marker, 14–100 kDa, TransGen Biotech Company, Beijing, China).

### Application of Abamectin and Measurement of OBP Expression Levels

To examine how moth tissues and the *PBP/GOBP* clade respond to chemical stress (insecticide), 4-day-old male adult silkworm *B. mori* were dipped in abamectin (China Agricultural University Biological Technology Co., Beijing, China) diluted in water following the method described in Xuan et al. (2015). Xuan et al. (2015) established that *B. mori* responds to abamectin with an array of “chemosensory protein” genes. Precise conditions for the insect treatments with the specific insecticide and controls were as described by Xuan et al. (2015) for the induction of “CSP” genes. The abamectin concentration was 4.2 ppm. The biological reason for this concentration was to overcome the slow penetration of insect cuticles by avermectins B1a and B1b (Clark et al., 1994). This abamectin concentration (4.2 ppm) compares with the reported low LC50 values with sublethal effects on insects (Batiha et al., 2020). The dipping duration was 10 s. This dose- and time-treatment was linked to the upregulation of detoxification genes such as *CYP4G25, CYP6AE21, CYP6B29, CYP15C1*, and *CYP333A2* (Xuan et al., 2015). Three replications (3x *n* = 10) were maintained for both abamectin exposure and control in real-time PCR as described by Xuan et al. (2015). The fourth batch of D4 adult males (*n* = 40) was maintained for electrophoresis, immunodetection, and protein data. As in the study of “*CSPs*,” the dipping method was chosen to optimize the deposition of chemical insecticide molecules on the epidermis and more precisely assess gene expression simultaneously in multiple tissues (see **Figures 4**, **5** and Supplementary Figures 3–5).

In total, about 100 silkworms were cut into pieces using scissors and forceps about 6 h after the dipping experiment (Xuan et al., 2015). On the basis of tissue distribution and ontogeny of the PBP/GOBP clade in the silkworm moth, the main olfactory sense organs and metabolic tissues were dissected for gene expression data. In three replications for both abamectin exposure and control, antennae, head, legs, thorax, gut, and fat body were dissected and immediately frozen in liquid nitrogen. RNA/cDNA samples were prepared as described under quantitative real-time PCR. In the fourth batch, epidermis and wings were added to the analysis but did not show any PBP/GOBP signals.

Protein samples from the antennae, head, legs, thorax, gut, fat body, epidermis, and wings of D4 adult virgin males treated with insecticide or control were prepared as described under protein analysis. Protein samples (1 mg/ml) for each tissue in the control and treated groups of D4 silkworms were analyzed by SDS-PAGE and Western blot using BmorPBP1, BmorGOBP1, and BmorGOBP2 antibodies as described before.

The qRT-PCR method was used to more precisely address *PBP/GOBP* gene expression and other gene protein families in response to chemical stress comparing sensory (antennae, head, and legs) and metabolic tissues (gut, thorax, and fat body). For qRT-PCR, messenger RNA samples were used to quantify *PBP1, PBP2, GOBP1, GOBP2, CYP306A1, CYP4M9, AOX1, AE41, JHE, cJHBP, hJHBP, EcR-B1*, and *OR1* gene expression in response to abamectin chemical as described in Xuan et al. (2015).

### Structural Modeling and Ligand Docking

The 3D models for BmorPBP1 (1DQE_mono2; X-ray, pH 8.4, resolution 1.8 Å) and BmorGOBP2 (2WCH; X-ray, pH 8.5, resolution 1.7 Å) were built using Modeler in Linux (Sali, 2020). For each of the two targets, structural models displayed 100% homology with templates from the Protein Data Bank (Sandler et al., 2000; Zhou et al., 2009). Docking and binding mode prediction of pheromone and non-pheromone ligands on PBP1 and GOBP2 were done with PyMOL and Vina (AutoDock Vina 4.2; Seeliger and de Groot, 2010; Trott and Olson, 2010) on “flexible protein”: 100 conformations for each protein were generated with Rosetta stimulating flexibility (Loshbaugh and Kortemme, 2020). Relative affinity in Kcal/mol corresponded to the best energy score of the most populated cluster using a contact-based ligand clustering approach for the identification of “active” compounds in *in-silico* screening (Mantsyzov et al., 2012). The root-mean-square deviation (RMSD) among ligand positions was < 2 Å.

First, we checked for the position of the bombykol molecule on the model to validate the method as performed by Klusák et al. (2003). For PBP1, the bombykol position is such that the hydroxyl group of the pheromone interacts with Ser56. For GOBP2, the bombykol position is such that the hydroxyl group of the pheromone falls close to Arg110 and Glu98 (Sandler et al., 2000; Zhou et al., 2009). We measured ΔG = −7.4 Kcal/mol for bombykol bound to PBP1 using Linux (see Supplementary Figure 6). This is consistent with actual *in vitro* ligand binding studies: ΔG = −8.1 Kcal/mol (Sandler et al., 2000; Campanacci et al., 2001; Leal et al., 2005; Mansurova et al., 2009; Supplementary Figure 6; Zenodo dataset). However, we measured a much lower relative affinity for bombykol bound to GOBP2 using Linux. The interaction of GOBP2 with bombykol could be due to the presence of water molecules in the protein binding site in the ligand-binding study *in vitro* (Zhou et al., 2009). The presence of a water molecule in the vicinity of Arg110 and Glu98 is favorable to the interaction of bombykol with GOBP2 (*In vitro*/Kd: 7.71E-06 ± 3.61E-06 vs. Linux/Kd: 4.27E-05 ± 4.38E-06 without water molecule in the vicinity of Arg110 and Glu98). So, in our docking experiments, bombykol achieved much more higher affinity for BmorPBP1 than for BmorGOBP2, which provides a greater degree of confidence in our modeling analyses based on bombykol for PBP1. We then used the same approach to measure the ability of non-semiochemical ligands to displace the bombykol molecule and integrate fully into the functional binding site of the protein (see **Figures 6**, **7** and Supplementary Figures 6–8 and Tables 1, 2 and Supplementary Tables 2, 3). Docking experiments were conducted using both BmorPBP1 and BmorGOBP2 as protein structures tested for binding non-semiochemical ligands such as vitamins (A, B1, B2, B3, B5, B6, C, D2, E, H, K1, K2, and K3), juvenoids (juvenile hormones I, JH II, JH III, and methoprene), regulatory neurotransmitters (acetylcholine and octopamine), methylxanthine drugs active on the nervous system and degraded by cytochromes (such as caffeine), insecticides (imidacloprid, pyrethrin II, and malathion), and several esters of carboxylic fatty acids important for various primary metabolic pathways, such as those of glucose and chemical energy (ethyl carbamate or urethane, dimethyl malonate, propionate, and succinate; Supplementary Figure 7).

**Table 1:** Binding energy scores of the interaction of BmorPBP1 and BmorGOBP2 protein structures with “non-semiochemical” ligands in docking experiments (Linux).

**“Non-semiochemical” ligand**	**ΔG (Kcal/mol) BmorPBP1**	**ΔG (Kcal/mol) BmorGOBP2**
Ergocalciferol	−11.8	−9.5
Vitamin K2	−11.8	−10.8
Vitamin K1	−11.5	−9.1
Vitamin E	−11.3	−6.6
Vitamin A	−10.9	−9.5
Riboflavin	−10.1	−6.2
Pyrethrin II	−9.5	−7.5
Vitamin K3	−9.6	−8.6
Juvenile hormone I	−9.4	−8.1
Methoprene	−9.1	−8.2
Juvenile hormone II	−9.0	−7.9
Juvenile hormone III	−8.9	−8.1
Imidacloprid	−7.8	−7.2
Thiamine	−7.0	−6.9
Biotin	−6.7	−6.1
Caffeine	−6.2	−6.1
Malathion	−6.1	−4.0
Dimethylmalonate	−5.6	−5.6
Pyridoxine	−6.2	−5.5
Nicotinamide	−6.1	−5.2
Nicotinic acid	−6.0	−5.1
Octopamine	−5.9	−5.7
Panthothenic acid	−5.9	−5.4
Ascorbic acid	−5.6	−5.1
Succinate	−4.8	−4.5
Acetylcholine	−4.7	−4.0
Ethyl carbamate	−3.6	−3.3
Propionate	−4.0	−3.3

*Those in red show predicted high affinity of PBP1 for vitamin compounds*.

**Table 2:** Main interactions (amino acid residues) between vitamin K1 and BmorPBP1 (pdb: 1DQE).

**Index**	**Residue**	**Amino** **Acid**	**Distance (Å)**	**Ligand** **Atom**	**Protein** **Atom**
1	12B	Phe	3.36	2,105	187
2	12B	Phe	3.48	2,109	185
3	12B	Phe	3.45	2,103	188
4	12B	Phe	3.65	2,111	182
5	36B	Phe	3.47	2,106	562
6	36B	Phe	3.65	2,104	563
7	37B	Trp	3.08	2,108	584
8	52B	Ile	3.94	2,105	817
9	52B	Ile	3.87	2,104	819
10	68B	Leu	3.77	2,124	1061
11	68B	Leu	3.57	2,118	1062
12	73B	Ala	3.72	2,118	1133
13	76B	Phe	3.59	2,114	1180
14	90B	Leu	3.95	2,115	1387
15	90B	Leu	3.51	2,117	1385
16	91B	Ile	3.91	2,118	1405
17	118B	Phe	3.59	2,102	1796
18	118B	Phe	3.59	2,119	1799
19	135B	Val	3.82	2,106	2067

*B means hydrophobic interactions. Amino acid Ser56 side chain (polar charges) conjugates with menadione (PyMOL and Autodock/Vina; Seeliger and de Groot, 2010)*.

Given all the ligands tested, many molecules representing all major chemical classes were subject to protein structure-ligand interaction by systematic docking (Supplementary Figure 7). For each “non-semiochemical” ligand, the interaction was measured with scoring of poses, motifs, accuracy metrics, model performances, and binding energy (ΔG in Kcal/mol) in protein docking for BmorPBP1 and BmorGOBP2 (Supplementary Tables 2, 3), identifying vitamins as potentially active “non-semiochemical” ligands for PBP1. The relative binding value was determined by docking using flexible protein in Linux. Linux yielded molecular protein models with a large hydrophobic pocket as observed in the X-ray structure (PDB: 1DQE). In addition, a qualitative analysis of the residues involved in the interaction with ergocalciferol, and vitamins A, E, K1, K2, and riboflavin were performed using the LigPlot+ software (Laskowski and Swindells, 2011).

## Results

### Age Variations of Male Pheromone Responsiveness in *B. mori*

Under our laboratory conditions (23°C, 70% relative humidity, 15-h light: 9-h dark), pheromone detection with young silkmoth resulted in the initiation of mating immediately after emergence.

**Figure 1:**
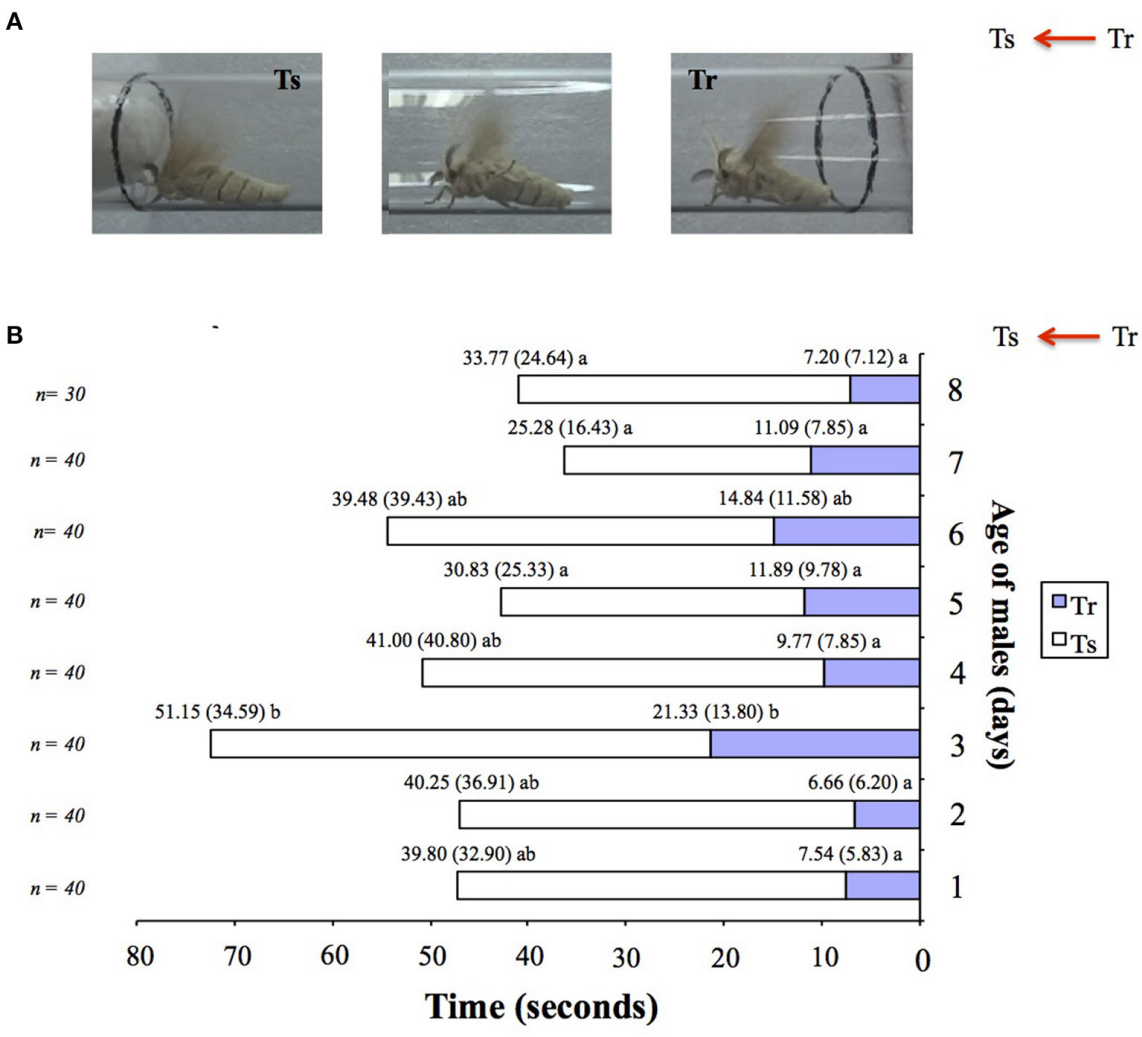
Age-related variations in pheromone responsiveness of male moths in *Bombyx mori*. **(A)** Behavioral responses of adult virgin male silkworm moths in an I-olfactometer to one calling Day 1 virgin conspecific female (odor source). Two-time responses (in s) are recorded: 1° Tr (time to react), 2° Ts (time to reach the source). **(B)** Behavioral responsiveness of 1-day-old (Day 1) to 8-day-old (Day 8) adult virgin male silkworm moths in the I-olfactometer (**A**) to one calling Day 1 virgin conspecific female (odor source). 0 was noted as the time when the males entered the upper part of the I-olfactometer. Average values and standard deviations (in brackets) are indicated atop the bars. Different letters indicate significant statistical differences at *p* < 0.001 by Mann–Whitney U test. n, number of males tested per age category.

By I-tube behavioral tests, we observed a consistent behavioral response through days 7–9 of adult male life. Time to react (Tr) and time to reach the source (Ts) were on average at about 11.29 ± 8.75 and 37.7 ± 31.38 s in 1- to 8-day-old males (n = 30–40; Figure 1). A few 9-day-old males tested were able to respond to pheromone (n = 10; Tr: 10.33 ± 6.51, Ts: 31.67 ± 20.55). The late-stage males (~D16–18) showed no response in the behavior test. They showed symptoms of disease, i.e., bluish-gray melatonin spots on the body starting on the abdomen.

### Expression of PBP and GOBP Genes in Aging *B. mori*

**Figure 2:**
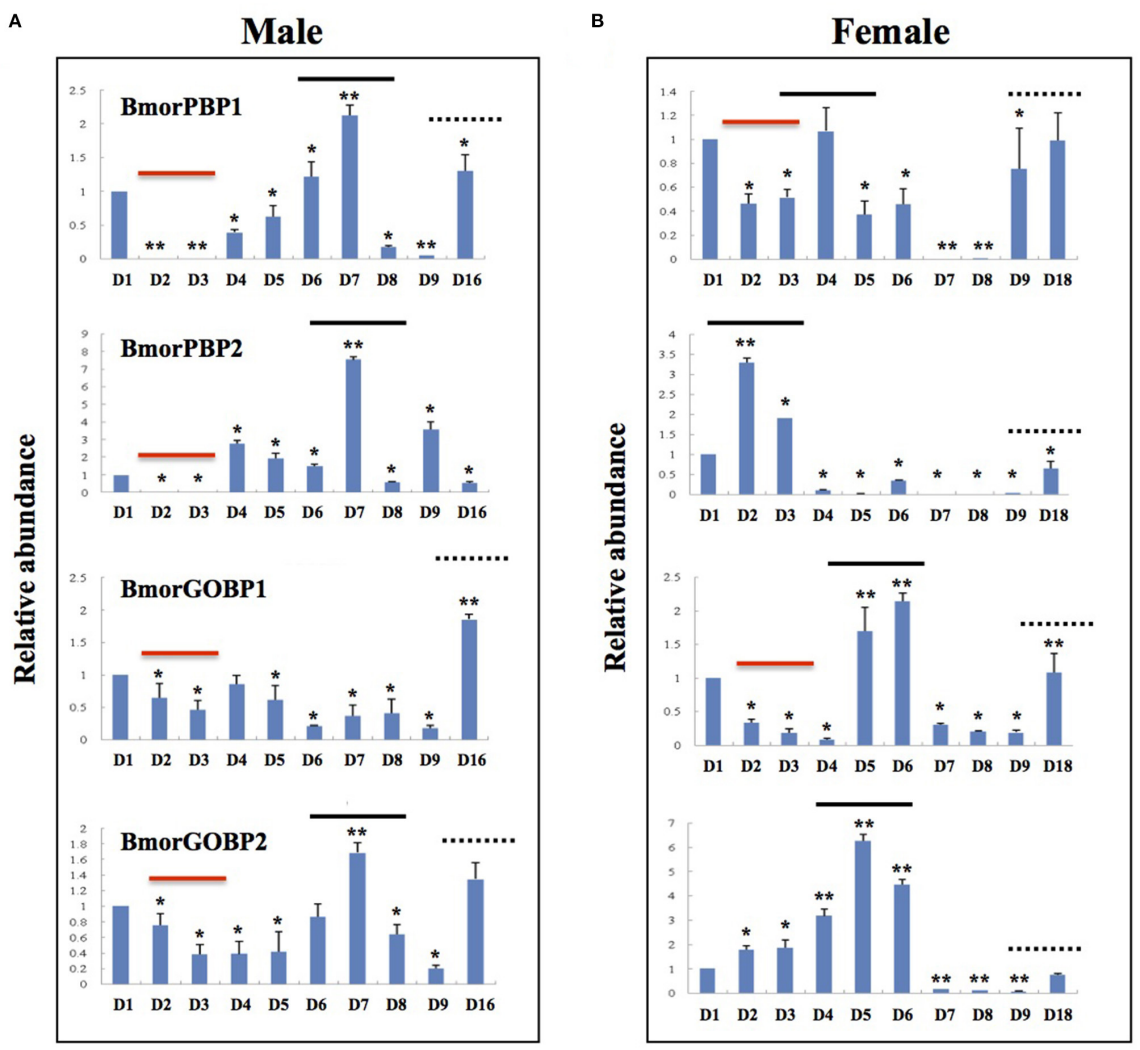
Age-related variations in the expression of *PBP1, PBP2, GOBP1*, and *GOBP2* genes in adult antennae of *Bombyx mori*. Quantitative real-time PCR (qRT-PCR) analysis of antennal expression levels of *BmorPBP1, BmorPBP2, BmorGOBP1*, and *BmorGOBP2* in different age groups of adult silkworm moths. Age variation of *PBP/GOBP* expression in **(A)** males and **(B)** females. Antennal gene expression levels in D1 (1-day-old) moths were used as reference (=1). The bold lines show age-dependent changes in *PBP/GOBP* gene expression levels in both sexes. The dotted lines show the upregulation of gene expression in the antennae of elderly moths (D9–D16/18). The red line shows the downregulation of gene expression in the antennae of young moths (D2–D3). The bars show means ± standard deviation (*n* = *9*). ^*^significant statistical differences at *alpha* = 0.05 by one-way ANOVA. ^**^significant statistical differences at *alpha* = 0.01 (ANOVA).

A real-time qPCR analysis of the expression profiling of the *BmorPBP1, BmorPBP2, BmorGOBP1*, and *BmorGOBP2* genes in the antennae across the whole adult lifetime of the silkworm showed that *PBP* and *GOBP* expression varied with age (Figure 2).

Analyzing *OBP* expression across ages compared with d1, expression peaks were noted for *PBP1, PBP2*, and *GOBP2* in 7-day-old males (Figure 2A), while different peaks of expression were observed in females (Figure 2B). Compared with d1, *PBP1, PBP2, GOBP1*, and *GOBP2* expression was reduced in d2 and d3 males (Figure 2A), while only *PBP1* and *GOBP1* expression was reduced in 2- and 3-day-old females (Figure 2B).

The expression of both *PBP* and *GOBP* was found to increase with age in both males and females (Figures 2A,B). *PBP* and *GOBP* expression increased in late-stage adults, but the females showed more gene changes than the males (D16–D18; Figures 2A,B). *PBP1, GOBP1*, and *GOBP2* expression increased in late-stage adult males. The four genes were induced over aging in the late-stage adult females (Figures 2A,B).

Analyzing the gene expression ratio for each day using *PBP1* as a reference showed about a 100-fold increase on Days 2 and 3 in *GOBP1* and *GOBP2* expression in the males but not in the females (Supplementary Figure 1). The *PBP1*-*PBP2* expression ratio was seen to change to 1:9 on Day 9 in males on the basis of gene expression using *PBP1* = 1 (Supplementary Figure 1A). *GOBP* gene expression peaked on Day 7 in the females (Supplementary Figure 1B).

### Expression of PBP and GOBP Proteins in Legs of Aging *B. mori* Females

Behavioral analysis showed no age-dependent variations in the responsiveness of males to the female sex pheromone in the silkworm moth *B. mori* (see Figure 1). However, an RNA analysis showed age-dependent changes in the variance of *BmorPBP1, BmorPBP2, BmorGOBP1*, and *BmorGOBP2* expression in the antennae in both sexes (see Figure 2). To check for non-pheromone functions in the moth PBP/GOBP clade, we set out to determine which tissues other than the antennae express PBP and/or GOBP proteins.

We used a large repertoire of sensory and metabolic tissues from the adult stage. We examined 4-day-old *Bombyx* female tissues (eggs, epidermis, fat body, gut, legs, thorax, wings, and head without antennae) from 1 to 5, 10, 20, and 40 mg/ml of protein concentration and checked for the presence of PBP and/or GOBP in each concentrated protein sample by SDS-PAGE and Western blot, and using a specific antibody (Supplementary Figures 2A,B). Protein bands corresponding to 14–16 kDa soluble proteins were detected in particular in the (x20) leg samples (Supplementary Figure 2A). No cross-reactivity with the BmorGOBP1 antibody was observed (Supplementary Figure 2B); however, cross-reactivity with the BmorPBP1 antibody identified 14–16 kDa proteins in the head (lacking antennae) and leg tissue samples (Supplementary Figure 2B).

**Figure 3:**
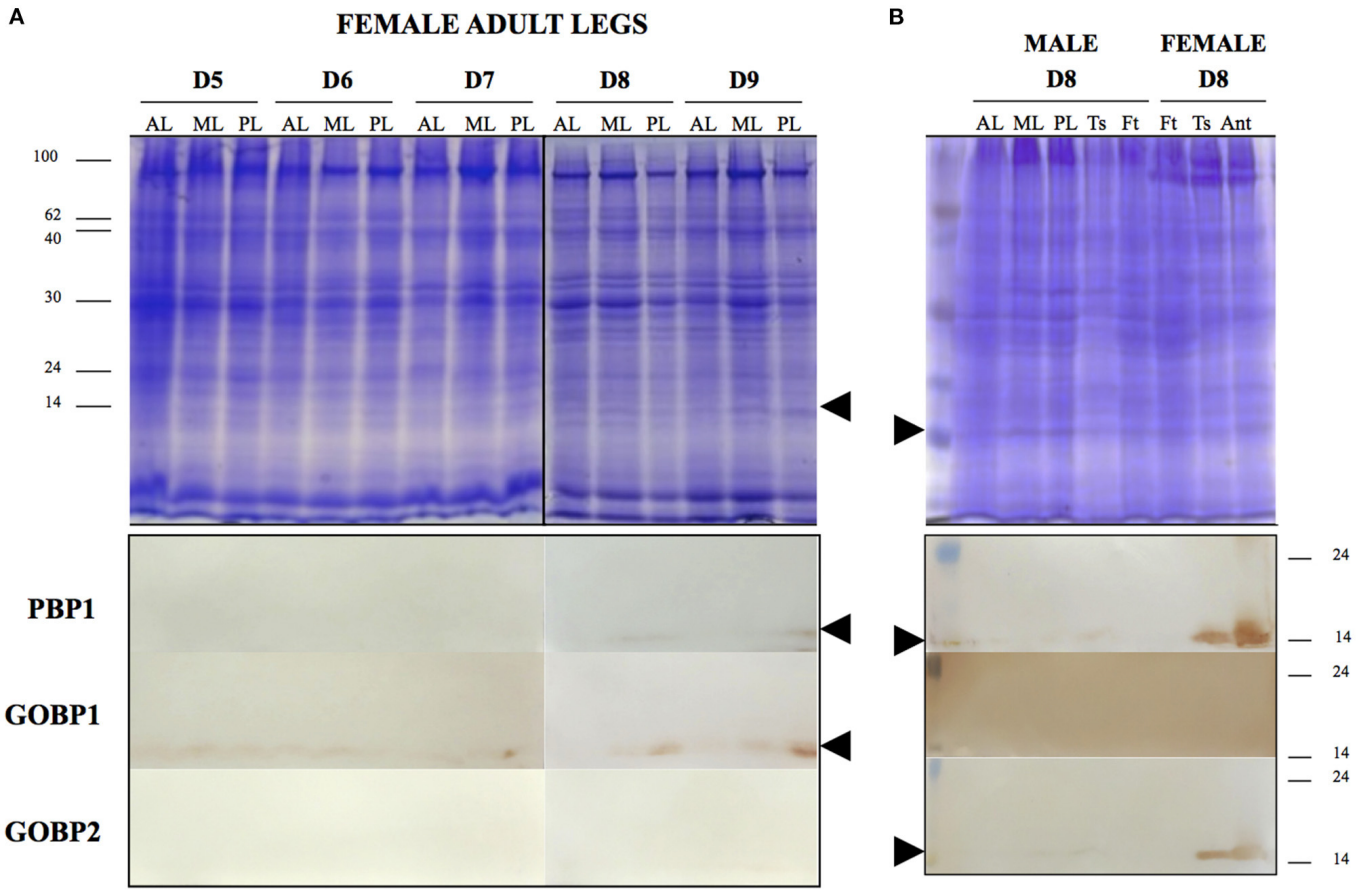
Age-related variations in the expression of PBP/GOBP protein in adult legs of *Bombyx mori*. **(A)** Gel electrophoretic separation (top) and immunoblots (below) of leg soluble protein samples in different age groups of female adult silkworms. AL, anterior legs; ML, median legs; PL, posterior legs. Age groups of female silkworm moths are from Day 5 (5-day-old, calling) to Day 9 (9-day-old, laying eggs). Below shows the results of female leg proteins exposed to BmorPBP1, BmorGOBP1, and BmorGOBP2 antibodies for immunodetection. The arrow tips show the position of PBP/GOBP-immunoreactive proteins (~16 kDa) in D7–D9 legs of mulberry adult female silkworm moth *B. mori*. **(B)** Gel electrophoretic separation (top) and immunoblots (below) of leg protein samples in the D8 group of male and female adult silkworms. For comparison between male and female, soluble proteins from the three pairs of legs (AL, anterior legs; ML, median legs; and PL, posterior legs) and different parts of the leg (Ts, tarsi and Ft, femur tibia) are exposed to BmorPBP1, BmorGOBP1, and BmorGOBP2 antibodies, respectively. The arrow tips show the position of PBP/GOBP-immunoreactive proteins (~16 kDa) in D8 leg tarsi of mulberry female silkworm *B. mori*. The same immunoreactive proteins are detected in the female antennal samples used as control (Ant).

Young (4-day-old) female leg samples with a very high concentration of protein (20 mg/ml) showed a signal when incubated with either the PBP1 or the GOBP2 antibody (Supplementary Figure 2). We then conducted a Western blot to investigate the abundance of the BmorPBP1, BmorGOBP1, and BmorGOBP2 proteins in leg tissues from females of different ages with only 1 mg/ml of protein concentration (Figure 3). Total protein concentration and SDS-PAGE (no apparent differences) were used as two loading controls (Figure 3A). We found that the BmorGOBP1 antibody labeled all the different female leg samples from D5 to D8. Interestingly, the GOBP1 immunoreactivity of the adult silkworm legs increased in an age-dependent manner. The BmorGOBP1 antibody more strongly labeled 14–16 kDa proteins in particular in posterior legs of the 9-day-old virgin females (see D9; Figure 3A). The PBP1 labeling resulted in a much weaker signal in the D8–D9 female (posterior) legs. No labeling or GOBP2 protein was evident in the legs of Days 5–9 female adults using samples with a low concentration of protein (1 mg/ml; Figure 3A). In another experiment, we used 1 mg/ml femur/tibia and tarsi samples in order to compare PBP/GOBP expression between the two sexes and different parts of the legs from unmated D8 female *Bombyx* (Figure 3B). The labeling of PBP/GOBP in the antennae was used as a control (Figure 3B). No labeling was found for GOBP1 in the antenna and leg samples (Figure 3B). We found weak labeling for PBP1 and GOBP2 in the male legs, but BmorPBP1 and BmorGOBP2 strongly labeled the leg tarsi from aging (8-day-old) females (Figure 3B). Therefore, our results from age-related changes in protein expression and immunoblots in the female silkworm moth *B. mori* are such that tarsi may turn GOBP1 off when PBP1 and GOBP2 are turned on D8. It may be that the expressions of GOBP1 and PBP1/GOBP2 are inversely related, suggesting a potential reciprocal regulation of transcription and/or translation in sex-, tissue-, and age-dependent manner (see Figures 2, 3).

However, the immunoblots showed that the expression of PBP/GOBP proteins was not specific to antenna and leg appendages (see Supplementary Figure 3). When performing other immunoblots using various male and female tissues, we found some immunoreactive signals for BmorPBP1 in the eyes, cephalic capsule, and whole insect head (brain and epidermis of the scalp, head without antenna). The heads of moths of both sexes expressed the BmorPBP1 protein (Supplementary Figure 3), while the BmorGOBP1 protein was found in the meconium (metabolic waste products from the pupal stage; Supplementary Figure 3).

### Expression of PBP and GOBP Genes in Non-sensory/Metabolic Tissues Across Different Developmental Stages of the Silkworm *B. mori*

**Figure 4:**
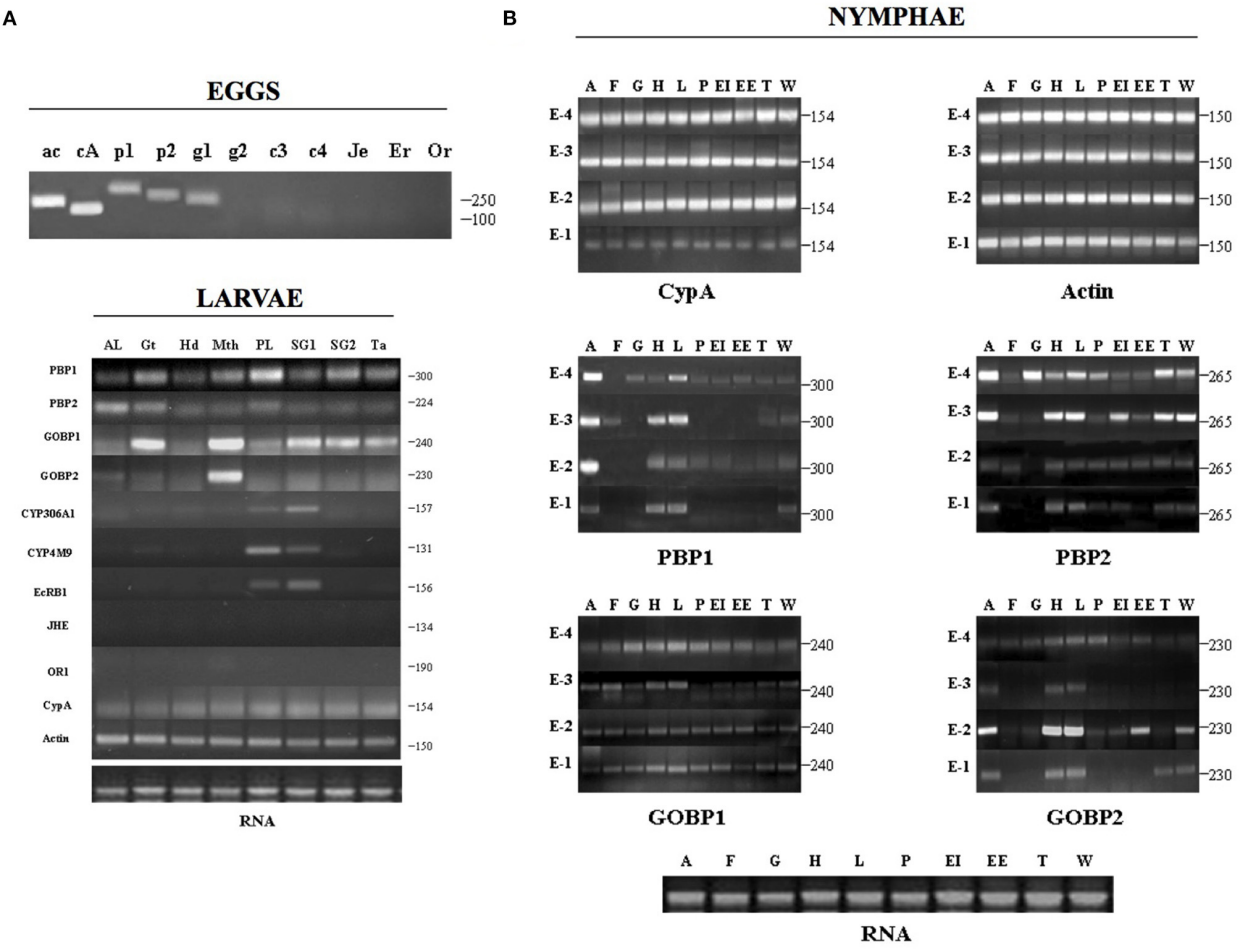
Ontogeny of *PBP/GOBP* expression. Expression of *BmorPBP1, BmorPBP2, BmorGOBP1, and BmorGOBP2* across 19 distinct tissues in *Bombyx mori* development. **(A)** Gene expression profiling in eggs (embryo) and fifth-instar larval tissues (AL, anterior -thoracic- legs; Gt, gut; Hd, head; Mth, mouth parts; PL, prolegs; SG1, silk gland (secretory section); SG2, silk gland (storage sac); Ta, dorsal tail end spine) of the silkworm moth. Eggs/ac: *actin*, cA: *CypA* (*cyclophilin A*), p1: *BmorPBP1*, p2: *BmorPBP2*, g1: *BmorGOBP1*, g2: *BmorGOBP2*, c3: *CYP306A1*, c4: *CYP4M9*, Je: *JHE*, Er: *EcR-B1*, Or: *OR1*. RNA: RNA control of larval tissue samples (1 μg/lane). The numbers indicate the length in bps of specific RT-PCR products. *Actin* and *cyclophilin A* are used as housekeeping control genes that are expected to express equally across the different tissues. **(B)** Gene expression profiling in late pupal stages (E-4 to E-1) of the silkworm. Using primers for *actin* and *cyclophilin A* (reference genes) shows an amplicon of expected length in similar amounts in all the tissue samples tested [A: antennae, F: fat body, G: gut, H: head (without antennae), L: legs, P: pheromone gland, EI: internal envelope (epidermis), EE: external envelope (cuticle), T: thorax, W: wings]. The numbers indicate the length in bps of specific RT-PCR products for *BmorPBP1, BmorPBP2, BmorGOBP1*, and *BmorGOBP2*, showing that RNA signals peaked not only in the antennae but also in many various metabolic tissues from E-4 to E-1. RNA: total RNA control of pupal tissue samples (E-1 to E-4: 1 μg/lane).

By SDS-PAGE/immunoblot and using protein samples of various adult tissues, the expression of PBP and GOBP was found to be not restricted to the antennae (see Figure 3 and Supplementary Figures 2, 3). The expression was evident in the tarsi of aging females seeking oviposition (Days 8–9; Figure 3). Protein expression data for Day 8 adults also showed rather convincing evidence for the presence of PBP1 in male (traces) and female heads without antenna and more abundant expression in cephalic capsules and in compound eyes, although no quantitative statements were possible because of lack of a loading control other than Coomassie Blue staining (Supplementary Figure 3). However, traces of GOBP were also detected in the meconium excreted by silkworm adults, suggesting a role in metabolic processes associated with insect development (Supplementary Figure 3). To investigate in detail the ontogeny of *PBP*/*GOBP* expression in *B. mori*, we used molecular biology methods and performed a much more comprehensive and specific detection of RNA transcripts to examine the expression profiles of *PBP1, PBP2, GOBP1*, and *GOBP2* from eggs to most of all the tissues developed in fifth instar larvae and E-5 through E-1 pupae (Figure 4 and Supplementary Figure 4).

Amplification of the two control genes (*actin3* and *cyclophilin A*) indicated the overall RNA integrity of the samples assayed. Semi-quantitative one-step RT-PCR amplification revealed the presence of transcripts for *PBP1, PBP2*, and *GOBP1* in the eggs. No PCR products were detected in the eggs for *CYP306A1, CYP4M9, EcRB1, JHE*, and *OR1* (Figure 4A). In the larvae, amplicons for *BmorGOBP1* expression were readily detectable not only in the mouthparts, gut, silk gland, and tail spine but also in both the thoracic and abdominal prolegs, although in lower amounts (Figure 4A). In the silk gland, *BmorGOBP1*-amplicons were noted in both the secretory section (rich in fibroin) and the storage sac (containing the gel-like unspun silk). *BmorGOBP2*-amplicons were found associated with the mouthparts, but *BmorPBP1*-amplicons were detected in all the larval tissues examined, including more particularly the gut and the prolegs (Figure 4A). Correlatively, the expression of ecdysone-related genes, such as *CYP306A1, CYP4M9*, and *EcRB1*, was found to be expressed in the prolegs and the silk gland of *Bombyx* larvae, while the *OR1* and *JH esterase* genes were silent (Figure 4A).

In addition, we detected PCR products for *BmorPBP1* in many various tissues, including not only the antennae but also the head (epidermis and brain) and legs across various pupal stages (Figure 4B). PCR products for *BmorPBP2* were found to be particularly high in the antennae and in the gut, head, legs, silk gland, thorax, and wings of E-4 and E-3 pupae (Figure 4B). *BmorGOBP1*-PCR products were particularly high starting at the E-4 pupal stage in many and various tissues (Figure 4B). Analyzing tissues from the pupae, the expression of *BmorGOBP2* was found to be not restricted to the antennae but also in the head and legs, in particular, 2-days before emergence (Figure 4B).

High levels of *BmorPBP1* expression were detected in the adult stage on the first-day post-emergence (Day 1). However, *BmorPBP1* expression on Day 1 was not restricted to the antennae, it was also detected in the head, legs, and epidermis (Supplementary Figure 4). Similarly, PCR products for *BmorGOBP2* expression in older adults (Day 9) were also found in various tissues, including the gut, head, legs, epidermis, and wings (Supplementary Figure 4). In the immature pupal stage (E-5, when tissues are colorless and soft), no PCR products or amplicons for *BmorPBP1* expression were detected. However, amplicons for *BmorGOBP1, BmorGOBP2*, and *BmorPBP2* expressions were detected rather ubiquitously in all the tissues investigated (Supplementary Figure 4), strongly suggesting a function related to early pupal development and tissue formation for these genes.

A BLASTn analysis in Silkbase (brain, early embryo of strain p50T, early embryo of strain N4, fat body, internal genitalia, midgut, anterior silk gland, middle silk gland, and epidermis) confirmed that transcripts encoding PBP1, PBP2, GOBP1, and GOBP2 are not restricted to the adult tissue, but found in different stages of the development, including embryo (Supplementary Table 1). By the BLASTn analysis in Silkbase, *BmorPBP1* expression was detected in the brain tissue and early embryo of the two *B. mori* strains (p50T and N4; Supplementary Table 1). *BmorPBP2* gene expression was detected not only in the brain and early embryo of p50T and N4 but also in the internal genitalia, similar to *BmorGOBP1* (Supplementary Table 1). However, *BmorGOBP1* expression was also detected in the anterior silk gland (Supplementary Table 1). *BmorGOBP2* expression was detected in the brain and early embryo RNA seq libraries, similar to *BmorPBP1* (Supplementary Table 1), strongly suggesting non-pheromone and/or non-olfactory functions for all the four OBP protein genes.

### Expression of PBP/GOBP Genes in Response to Insecticide Exposure

**Figure 5:**
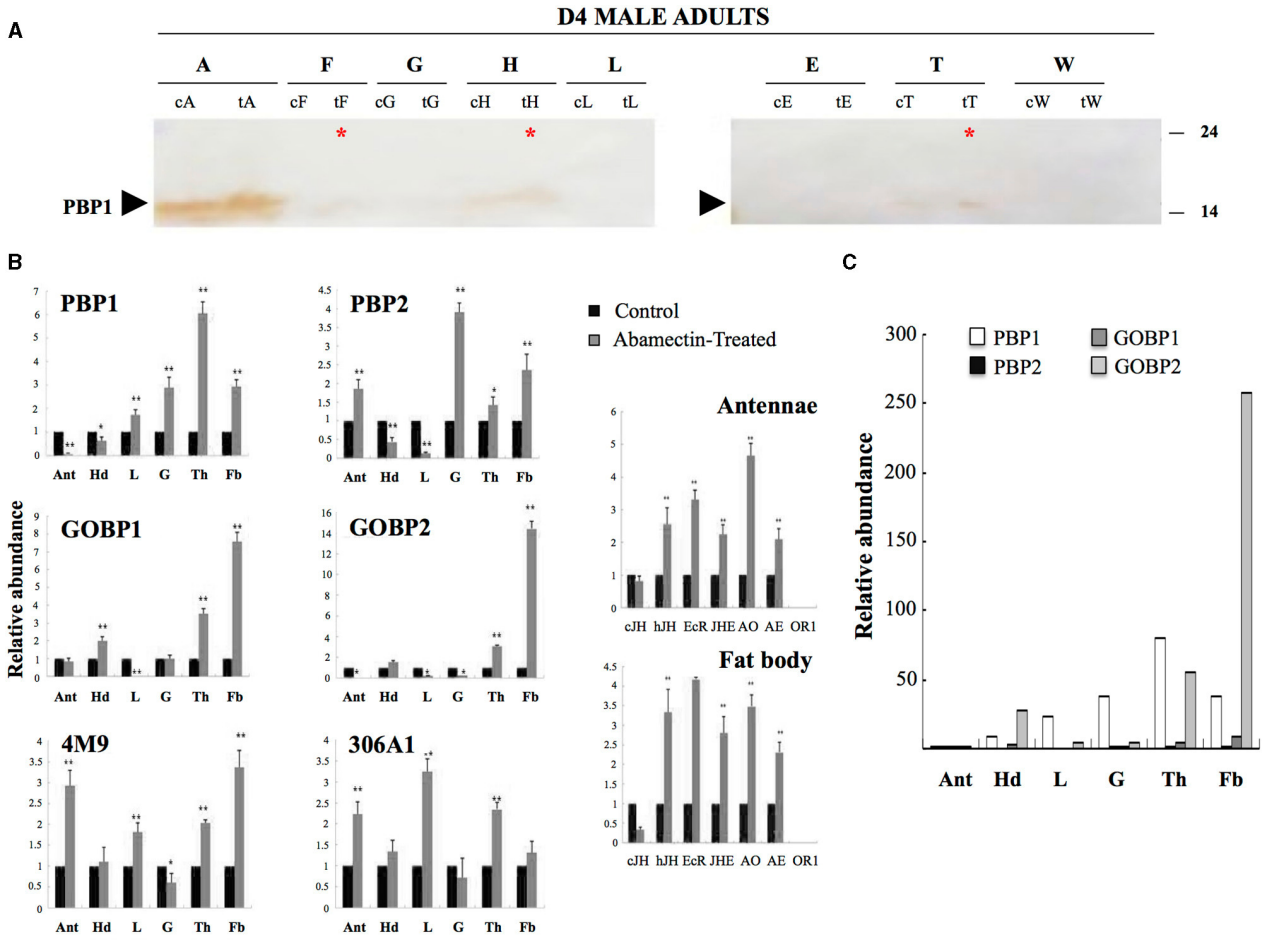
Induction of *PBP/GOBP* expression by abamectin. Tissue-specific regulation of *PBP/GOBP* in male adult *Bombyx mori* treated with insecticide. **(A)** Effects of abamectin insecticide on BmorPBP1 protein expression. Immunoblots of protein samples (1 mg/ml) from 4-day-old male adult tissues probed with antibodies against BmorPBP1. c, control; t, treated. The arrow tip indicates the position of PBP1 on the blot membrane. The asterisk shows PBP1 signals in various metabolic tissues in response to abamectin exposure, although in much lower amounts compared with the antennae (cA, tA). Antennae (A), fat body (F), gut (G), head without antennae (H), legs (L), epidermis (E), thorax (T), and wings (W). The arrow tip indicates the position of 16 kDa proteins on the gel. The numbers aside give the positions of protein molecular weight markers. **(B)** Quantitative real-time PCR (qRT-PCR) analysis of antennal (Ant), head (Hd), legs (L), gut (G), thorax (Th), and fat body (Fb) expression levels of *BmorPBP1, BmorPBP2, BmorGOBP1, BmorGOBP2, CYP4M9* (cytochrome P450 4M9), and *CYP306A1* (cytochrome P450 306A1) in the control (saline) and abamectin-treated groups of male adult silkworm moths. Control levels are used as reference (=1). On the right: qRT-PCR analysis of antennae/fat body expression levels of *cJHBP, hJHBP, EcR-B1, JHE, AOX1, AE41*, and *OR1*. CypA: cyclophilin A, cJH: cytosolic juvenile hormone binding protein (cJHBP), hJH: hemolymphatic JHBP, EcR: ecdysone receptor variant B1 (EcR-B1), JHE: JH esterase, AO: antennal oxydase-1 (AOX1), AE: antennal esterase-1 (AE41), OR1: pheromone/olfactory receptor-1. The bars show means ± standard deviation (*n* = 9). The single asterisk ^*^significant statistical differences at *alpha* = 0.05 by one-way ANOVA. The double asterisk ^**^significant statistical differences at *alpha* = 0.01 (ANOVA). **(C)** Comparative *BmorPBP1, BmorPBP2, BmorGOBP1*, and *BmorGOBP2* gene expression profiles across different tissues in response to insecticide exposure in male adult silkworm moths. Focus on the x-fold increase in gene expression from RNA samples (gut, thorax, and fat body) in the same experiment than 5B (Step 1). Quantitative real-time PCR results with means (*n* = 9) of metabolic tissues compared with antennae (Ant) used as reference (Step 2: Ant expression = 1). Upregulation of PBP/GOBP expression in metabolic tissues after insecticide abamectin treatment.

We then applied a toxic macrolide insecticide endectocide molecule (abamectin) to check for the involvement of OBPs in insect defense following Xuan et al. (2015). Accordingly, RNA transcripts and the protein expression of *BmorPBP1, BmorPBP2, BmorGOBP1*, and *BmorGOBP2* were assessed in a comparative study of sensory and metabolic tissues from D4 silkworm males using qRT-PCR and immunoblot (Figure 5).

By immunoblot and using a group of tissues from 4-day-old *Bombyx* males, we found high expression levels and abundance of BmorPBP1 in the antennae (Figure 5A). However, in this immunoblot experiment, analyzing two different sets of samples (treated vs. control), we found that BmorPBP1 protein expression was not restricted solely to the antennae, and BmorPBP1 signals were also found in other tissues, such as fat body, head, and thorax, particularly in the group of males treated with the abamectin insecticide (Figure 5A), showing the increased synthesis of BmorPBP1 in metabolic tissues in response to chemical stress. However, in the blots (Figure 5A), it appeared that there was also a signal in the control samples for PBP1 in the head and thorax. Therefore, it cannot be said that the presence of PBP in these tissues is attributed only to stress response.

The qRT-PCR results showed that poisoning the tissues of male silkworm moths with an insecticide, such as abamectin, showed that abamectin exposure modulated the expression of all four genes examined depending upon tissue and gene (Figure 5B). For example, PBP1 and GOBP2 showed decreased expression in the antennae, but PBP2 showed increased expression. Similarly, PBP1/2 showed decreased expression in the head, but GOBP1 showed increased expression. In the legs, PBP2, GOBP1, and GOBP2 showed severely decreased expression, but PBP1 rather showed increased expression. So, it is not always the case for the four genes that the expression was decreased (Figure 5B). However, in some instances, as shown in Figure 5B, an increase in the expression is apparent.

Abamectin insecticide exposure led to the increased expression of the *BmorPBP1, BmorPBP2, BmorGOBP1*, and *BmorGOBP2* genes in various metabolic tissues, such as the thorax and fat body (Figure 5B). The exposure also led to the increased expression of *BmorPBP2* in the gut (Figure 5B). Similarly, abamectin promoted the increased expression of *BmorPBP1* in other tissues including not only the legs but also the gut, thorax, and fat body (Figure 5B). *BmorPBP2* gene expression was increased by a factor of 4 in the gut, while the two *B. mori GOBP* genes, *BmorGOBP1* and *BmorGOBP2*, were upregulated in the thorax and fat body, two organs for intermediary metabolism in the insect, by a factor of 4 to 16 in response to abamectin exposure (Figure 5B).

Intriguingly, applying abamectin by dipping the moth in the insecticide solution did not change the expression levels of the gene encoding olfactory pheromone receptor (*OR1*; low detection levels). However, exposure to abamectin upregulated not only the *PBP* and *GOBP* genes but also 20-hydroxyecdysone-related genes and cytochrome oxidase genes responsible for moth metabolism in the fat body (Figure 5B). In contrast, abamectin had no effect on the antennal expression of *cJHBP* or *OR1*. *PBP/GOBP* was stimulated along with *CYP306A1, CYP4M9, EcR-B1, AOX, AE, JHE*, and *hJHBP* in response to insecticide exposure (Figure 5B). *BmorPBP1* and *BmorGOBP2* expression increased by up to 50–300 times higher in metabolic tissues, such as the gut, thorax, and fat body, in response to abamectin exposure (Figure 5C), strongly suggesting a metabolic function for genes of the *PBP/GOBP* clade in moths.

### Docking of Non-semiochemical Ligands on PBP and GOBP Structures

**Figure 6:**
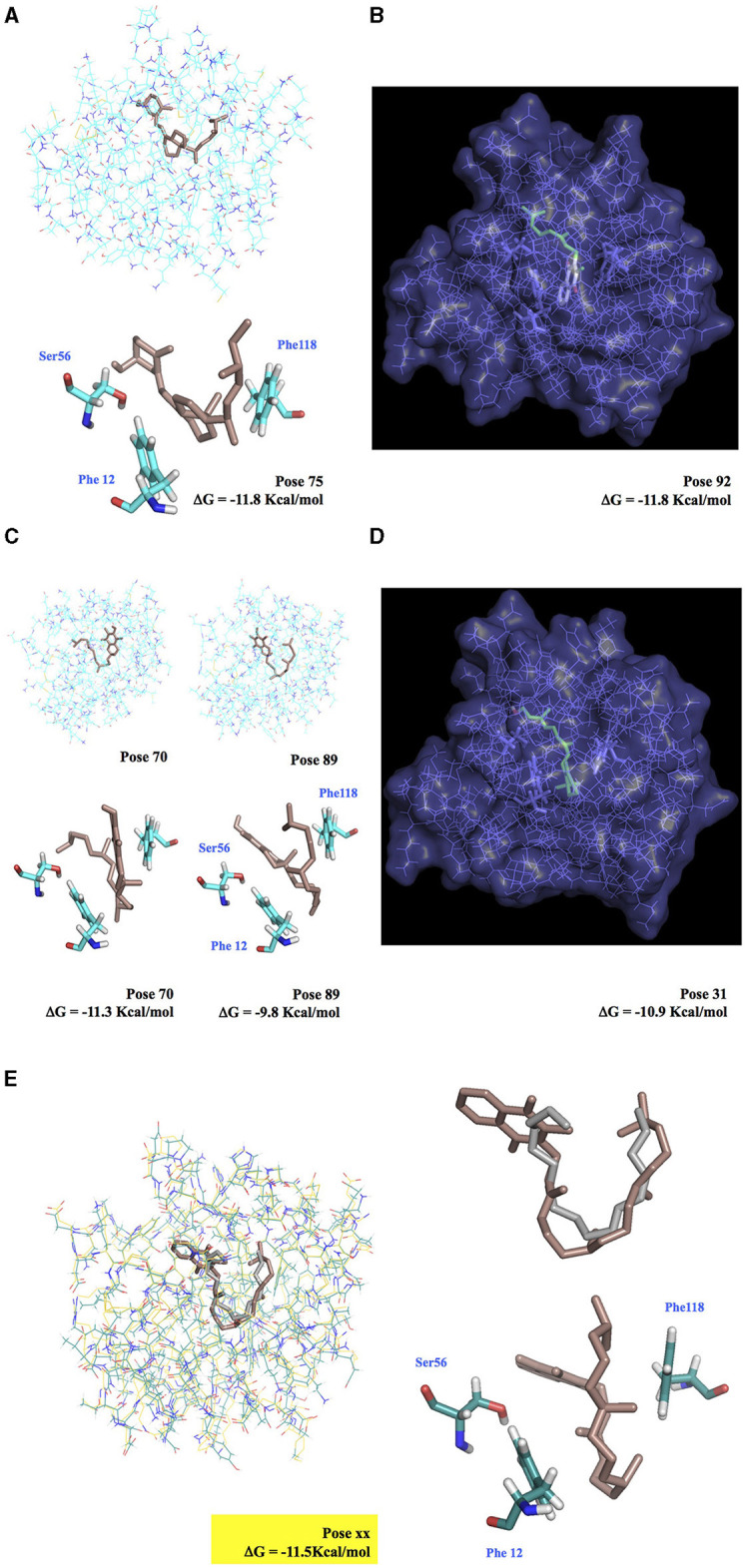
Docking simulation of vitamin molecules integrated into BmorPBP1 binding pocket. **(A)** Ergocalciferol-BmorPBP1 protein complex. **(B)** Cutaway view of the interaction of BmorPBP1 with vitamin K2. **(C)** Vitamin E-BmorPBP1 protein complex in two binding modes. **(D)** Cutaway view of the interaction of BmorPBP1 with vitamin A. **(E)** Interaction of BmorPBP1 with vitamin K1 (docking Vina, in brown) and bombykol (X-ray structure, in gray). Vitamin K1 completely overlaps with bombykol. Vitamin K1 attached to Ser56, Phe12, and Phe118 folds in the same position compared with bombykol in the PBP binding site. ΔG shows the relative binding affinity value for vitamin compounds to PBP1. Docking pose shows scored matching or “best-fit” of fragment atoms from vitamin compound and BmorPBP1 in a multiple-grid arrangement.

**Figure 7:**
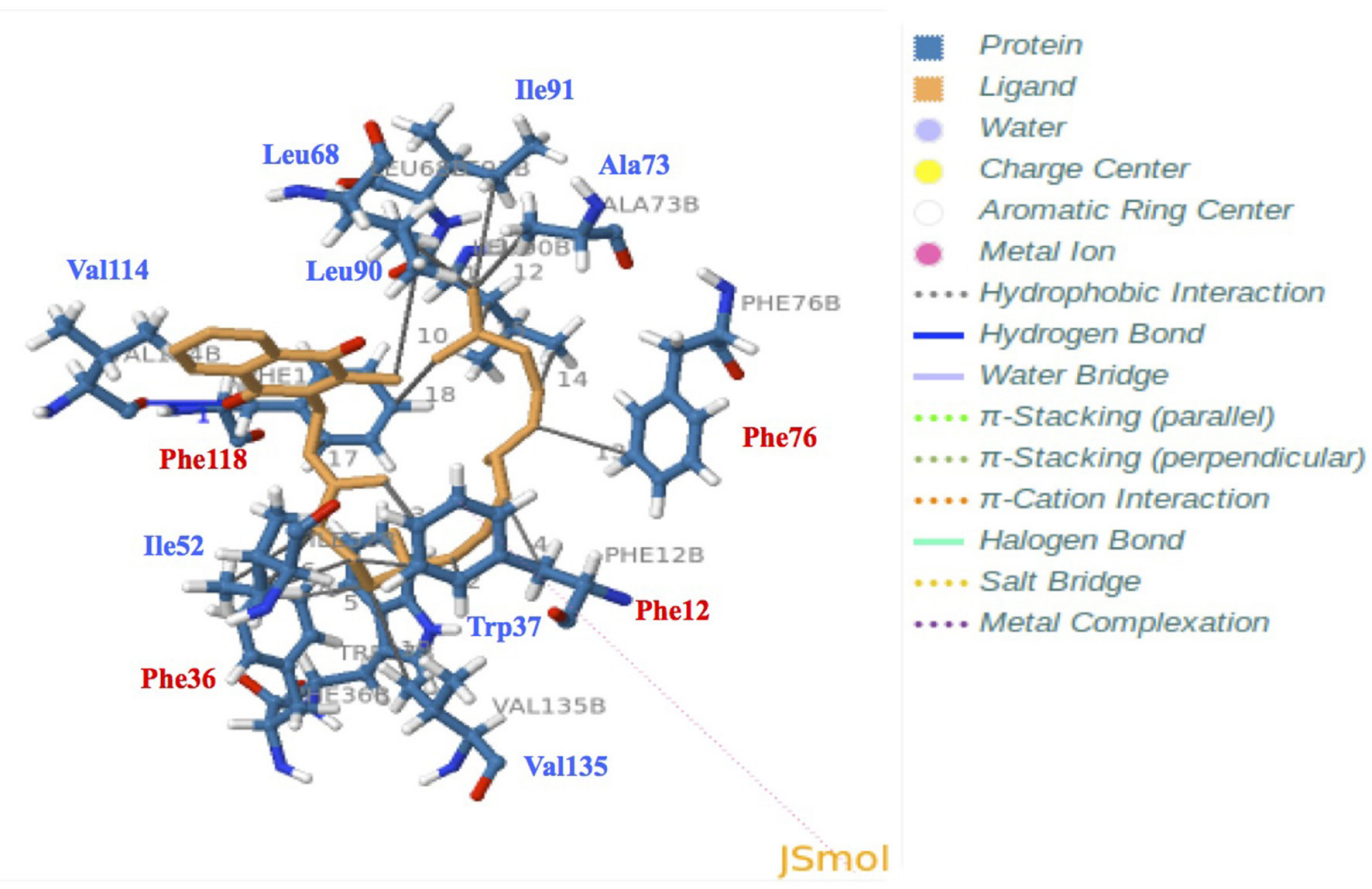
Docking and 3D analysis of BmorPBP1 internal cavity bound to vitamin K1 ligand molecule. Hydrophobic interactions: Phe12, Trp37, Ile52, Leu68, Ala73, Phe76, Leu90, Ile91, Phe118, and Val135. Hydrogen bonds: Index 1, Residue 114B, amino acid: Valine, distances H–A: 3.04 Å, distances D–A: 3.68 Å, donor angle: 124.05°, donor atom: 2,131 [O_3_], acceptor atom: 1,742 [O_2_]. The predicted vitamin K1-BmorPBP1 complex is visualized with JSMol (modality of Jmol: An Open-Source Java Viewer for Chemical Structures in 3D).

To justify the tissue-developmental profiles (see Figures 2–5 and Supplementary Figures 1–5, Supplementary Table 1), we tested 28 different non-semiochemical ligands, including insecticides, juvenoids, caffeine, esters of carboxylic fatty acids, and multiple vitamins in docking of BmorPBP1 and BmorGOBP2 binding sites using Linux for 3D modeling and AutoDock Vina (see Figures 6, 7 and Supplementary Figures 6–8, Tables 1, 2 and Supplementary Tables 2, 3, Zenodo dataset). First, we showed that the position of the bombykol molecule on the model was similar to that observed on the crystal structure. This was attested by a measurement of the binding energy values (in Kcal/mol). The binding energy values obtained with Linux were very close to those obtained with X-ray, e.g., for the measurement of bombykol bound to PBP1: ΔG = −7.4 vs. −8.1 Kcal/mol (Sandler et al., 2000; Campanacci et al., 2001; Leal et al., 2005; Mansurova et al., 2009; Supplementary Figure 6; Zenodo dataset). The binding affinity of bombykol with PBP1 was much higher than that measured for bombykol-GOBP2 (only 4.3 in the absence of water; Supplementary Figures 6, 8 and Supplementary Table 3; Zenodo dataset).

Among the different models, our docking study showed that the PBP1 binding pocket could interact directly with K1 (distance < 4 Å, ΔG = −11.5 Kcal/mol), and that K1 could be accommodated into the PBP1 binding pocket in the same seating U-shaped configuration compared with bombykol (Figures 6, 7 and Supplementary Figure 6 and Tables 1, 2, Zenodo dataset). Furthermore, the binding of K1 was very similar to the binding of bombykol in BmorPBP1 (Figures 6, 7 and Table 2). In contrast, BmorGOBP2 showed a different binding site for multiple various non-semiochemicals, such as vitamins (Supplementary Figure 8). Except for vitamin K2, relative energy values (ΔG) were < −10 Kcal/mol for most of all the ligands tested with BmorGOBP2 (Supplementary Figures 7, 8 and Table 1). The best affinity value was obtained when the ligand molecule (A, E, ergocalciferol, K2, or pyrethrin II) fell inside the central hydrophobic pocket of the protein, but this rarely happened with BmorGOBP2 (Supplementary Figure 8 and Supplementary Table 3, Zenodo dataset).

## Discussion

We have analyzed the behavioral responsiveness of the adult silkworm moth *B. mori* together with the tissue/development profiling of four odorant-binding proteins (referred to as BmorPBP1, BmorPBP2, BmorGOBP1, and BmorGOBP2) that represent the derived PBP/GOBP clade of Lepidoptera. The OBPs we studied are well-known, e.g., BmorPBP1 and BmorGOBP2, exclusively defined by their affinity to bombykol (Sandler et al., 2000; Zhou et al., 2009). However, the four main points in our study are new and innovative with a marked impact on research as follows: (1) the four OBPs exhibit an age-dependent expression that is independent of pheromone release/detection, suggesting roles outside of the olfactory paradigm, (2) the expression of moth PBPs and GOBPs in non-olfactory tissues in different developmental stages points to an alternative role of these proteins, (3) their expression can be induced under specific physiological conditions (chemical stress) by an insecticide, perhaps indicating that the alternative function is related to insecticide resistance, and (4) their ability to bind non-semiochemical ligands, such as vitamin compounds, is in agreement with the expression of *OBP* genes in bacteria and a great variety of metabolic organs and tissues during insect development.

First, by analyzing age-related behavioral responses of the male silkworm *B. mori* and *PBP*/*GOBP* gene expression in the male antennae by relative RNA abundance and quantitative real time-PCR, we note a reduction in the accumulation of PBP and GOBP RNA in the young males, a linear increase from D4 to D7 for *PBP1*/*GOBP2* and *BmorPBP1, BmorPBP2, BmorGOBP1*, and *BmorGOBP2* expressions in time or age. This corresponds to previously reported observations on *B. mori* of no or reduced sex pheromone communication, mating activity, and reproduction (Biram et al., 2005). D2–D3 age-related *PBP*/*GOBP* expression changes in the males are not associated with the decline in physical responsiveness or the neural discrimination of sex pheromone components (see Figures 1, 2). Then, we observe increased *PBP/GOBP* gene expression in the antennae of aging adult silkworm moths (see Figure 2 and Supplementary Figure 1), which could be directly linked to age-related behaviors and the activation of specific response control elements.

Interestingly, many response control elements are found upstream of the four *OBP* genes studied here. Although alternative promoters and/or regulatory DNA sequences could appear in more distant regions, these sequences may help to explain the *OBP* expression patterns observed in our study (see Figure 2 and Supplementary Figure 1). The mechanisms underlying the *PBP1*-*PBP2* vs. *GOBP1*-*GOBP2* expression, in particular, should be investigated with caution. The *PBP1, PBP2, GOBP1*, and *GOBP2* genes are localized in the same genomic DNA regions 7K−9K at the tip of chromosome 19 (Bm_Scaf100). In the silkworm, *PBP1* and *PBP2* are tandem genes (only separated by 859 bps), while *GOBP1* and *GOBP2* are separated by 115,673 bps and not so closely linked on the genetic map of the silk moth *B. mori* (KAIKObase; Liu and Picimbon, 2017).

In the *B. mori* genome, we find a signal transducer and activator of transcription (JF267349), a sericin promoter region (HQ702379), and multiple transposable elements (Bm1-450bp, BMC1, Hope, gypsy-Ty3-like Kabuki, LTR Yamato, Manga, Mariner, Minichikuri, microsatellite repeats, non-LTR_TREST-W, Rikishi marker, Suju^*^Minghu, Tama and TREST1) in front of the *PBP1*/*PBP2* (contig35963), *GOBP1* (contig35949) and/or *GOBP2* (contig35962) genes. Coincidentally, the expression profile of the *GOBP* genes differs in the sexes. The antennal expression of *PBP1* and *PBP2* is constant throughout early adult male life (i.e., D1-D8), whereas the expression of *PBP*/*GOBP* is only synchronous after D5 in the females (see Figure 2 and Supplementary Figure 1). This might indicate the occurrence of sex-specific regulation of *PBP*/*GOBP* clade expression, in particular, in the antennae of aging moths.

This view is supported further by the observation that *BmorPBP1, BmorPBP2, BmorGOBP1*, and *BmorGOBP2* are highly expressed in late*-*stage *B. mori* adults (about 2-week-old; Figure 2). The increased accumulations on Day 16/18 in the antennae when the insects are becoming more sensitive to chemical or microbial toxin infection perhaps mean that PBPs and GOBPs give rise to a specific phenotype (longevity). A high expression of these OBPs in late age moths might have phenotypic consequences, in particular, on lifespan. There are numerous gene expression hallmarks of cellular aging, such as dysregulation of immune system genes and signaling in eukaryotes from yeast to humans (Frenk and Houseley, 2018). However, the high expression levels of the *PBP* and *GOBP* genes in late-stage adult moths may coincide with the moth aging process, particularly in the absence of mating. The absence of mating can affect the endocrine system, female and male moth fitness, and senescence (Truman and Riddiford, 1971). The adult lifetime among many various silkworm strains is known to be genetically controlled (Choi et al., 2013). Therefore, it could be that the *PBP/GOBP* clade plays a crucial part in lifespan-related genes controlling neuronal plasticity, moth aging, and/or senescence in a *B. mori* strain, such as Qingsong x Haoyue (Münch et al., 2008; Jarriault et al., 2009; Jindra et al., 2013). Our results suggest genetic interactions between *PBPs*/*GOBPs* and other kinds of genes involved in phenotypic plasticity, resistance, and longevity in insects. Aging beyond the main reproductory period might be particularly relevant to seek additional matings and/or more suitable oviposition sites, especially in long-lived species of moths. The ability of female spruce budworms (Tortricidae, *Choristoneura*) to discriminate cues from host plants for oviposition is very bad in virgins, but changes markedly following mating (Wallace et al., 2004). Additionally, it is known that *OBP* expression levels can be significantly affected by mating in both sexes (Boni Campanini et al., 2017). Therefore, the expression peak of *OBPs* on D4–D7 and D9–D16/18 may be due to the activation of common promoter regulatory regions for “late” mating activity and/or oviposition behavior, although promoter complexity may decrease from immediate newly emerged early to late elderly senescence genes. Promoter regions and transposable elements responsible for the specific expression of senescence genes have been identified in various organisms (Noh and Amasino, 1999; Andrenacci et al., 2020). It would be interesting to identify which promoters (or retroposons) in front of *PBP*/*GOBP* genes are involved in aging silkworm moths. When “late” expression is synchronized for the four genes in the *PBP*/*GOBP* clade in females, but not in males (see Figure 2 and Supplementary Figure 1), there are usually sex-specific functions and gene regulatory processes expected for the clade in the antennal tissues of aging moths.

Very interestingly, we find sex- and age-dependent regulation of *PBP*/*GOBP* gene expression not only in the antennae but also in the legs from the silkworm moth (see Figure 3). This provides additional insights into sex-, age- and tissue-dependent regulation of genes within this OBP clade of Lepidopterans (see Figures 2, 3 and Supplementary Figures 1, 2). The expression of PBPs and GOBPs in the moth legs is an interesting result, but not surprising, for two reasons: (1) as the antennae and legs are appendages of a particular segment and have similar embryonic origins and (2) many other OBPs are known to be expressed in the legs of insects (Starostina et al., 2009; Yin et al., 2012; Sun et al., 2013, 2017; Ohta et al., 2015; Li et al., 2017, 2020; Guo et al., 2018; Huang et al., 2018; Zhang et al., 2018; Ozaki, 2019). Some insects have sensory hairs on the legs, particularly the tarsi and tibia of pairs of hind legs. It is possible that the OBPs expressed in the legs are implicated more in taste detection than in pheromone detection (Ozaki et al., 2011). However, not only the differential expression of PBP1/GOBP2 vs. GOBP1 but also the increased expression of OBPs in the legs of aging female silkmoths are a very surprising result (see Figure 3). This observation allows debating about PBP/GOBP clade and a function narrowly tuned to moth sex pheromone. In *B. mori*, females have four to seven times more tarsal sensilla than males (Takai et al., 2018). The males engage in orientation and locomotion behaviors during or close to mating that involves the legs and would require any sensilla on the legs to be protected from pheromone overdoses, but 8-day-old female silkworms need to engage in an oviposition behavior to lay eggs on the most suitable plant leaves and cocoons. The ontogenies of adult male and female silkworms with regard to the expression of the PBP/GOBP clade were not keyed to pheromone exchanges, mating behavior, and reproduction. We show what happens if males and females are reared separately and if an individual ages without mating in both sexes. It is known that pheromone and mating activities start immediately after eclosion and are terminated after 8–10-days in the adult life cycle of silkworm *B. mori* maintained in laboratory conditions. Females start to lay eggs without mating on day 8 in the same laboratory conditions (Ando et al., 1996; Matsumoto et al., 2002; Blomquist et al., 2012). Therefore, we find a correlation between oviposition behavior and OBP expression in the legs of silkworm moths. There is a difference in pre-oviposition (D5-D7) vs. post-oviposition (D8–D9; see Figure 3), suggesting an age-dependent increase in the activation of key regulatory elements in the front of *PBP/GOBP* genes in the legs of the moths. This difference was apparent not only for PBP1 but also for GOBP1. GOBP1 showed presence in all ages but significantly increased in the posterior legs of D8–D9 females (see Figure 3). The expression of PBP and GOBP in the tarsi of 8-day-old virgin females could suggest a taste gustatory function involved in host plant recognition for oviposition of the silkworm (Figure 3), as described for OBP11 in the alfalfa plant bug *Adelphocoris lineolatus* and specific taste receptors in the papilionid swallowtail butterfly *Pachlioptera aristolochiae* (Ozaki et al., 2011; Sun et al., 2016).

However, here, we observe the presence of BmorPBP1, BmorGOBP1, and BmorGOBP2 proteins not only in the forelegs but also in the cephalic capsule, compound eyes, and meconium of D8 adult silkworm moth *B. mori* (see Supplementary Figures 2, 3). Therefore, it is not only the antennae/legs appendages but also neurons in the brain and retina that seem to express the *PBP/GOBP* clade. Meconium is what remains from the gut following the process of metamorphosis from the pupal to adult digestive tract. So theoretically, there could be a lot of different molecules, such as PBP and GOBP, related to the physiology of this process. Accordingly, we have analyzed the ontogeny of *BmorPBP1, BmorPBP2, BmorGOBP1*, and *BmorGOBP2* gene expressions through all the various stages of *Bombyx* development from egg to late instar larvae and pupae. We report about the induction of gene expression in the *PBP/GOBP* clade much before the appearance of adult rigid organs (see Figure 4 and Supplementary Figure 4). We find *PBP1, PBP2*, and *GOBP1* expression in eggs (embryo; see Figure 4A and Supplementary Table 1) as found for *OBPs* in many various insect species, including particularly egg noctuid moths (Amenya et al., 2011; Sun et al., 2012). We find a low-abundance *GOBP2* sequence in *B. mori* eggs by analyzing the EST contain the NCBI library or database (HX266954). Therefore, the *PBP/GOBP* clade is not only expressed in adults but also in eggs (embryo), and this seems to be a marked expression throughout many different species of moths. We also found *BmorPBP1, BmorPBP2, BmorGOBP1*, and *BmorGOBP2* expression in numerous larval tissues, which is in good agreement with EST resource in NCBI where numerous hits for the two *GOBP* sequences can be found in tags of silkworm larvae libraries (*GOBP1*: FY38063-FY755241, *GOBP2*: FY741717-FY57625; see KAIKObase, Shimomura et al., 2009). Like EST-RNA sequences, RT-PCR products for *PBP1, PBP2, GOBP1*, and *GOBP2* genes do not necessarily signify the presence of the respective proteins, but they certainly signify the induction of the respective genes not only in the egg but also in many tissues of the larvae of the silkworm moth (see Figure 4). In the fifth instar larvae (feeding stage) of the silkworm *B. mori*, we find the induction of the *GOBP1* and *GOBP2* expression to be mainly associated with the mouthparts, confirming the studies of Vogt et al. (2002). However, our results show that *GOBP1* expression is not restricted to chemosensory sensilla surrounding the mouth, but that *GOBP1* RNA transcripts are also particularly abundant in the secretory section (rich in fibroin) and the storage sac (unspun silk) of the moth silk gland (see Figure 4A). This is in agreement with the BLASTn analysis of *B. mori* tissues in Silkbase. Based on BLASTn data of Silkbase (Supplementary Table 1), we emphasize the presence of PBP and GOBP RNA sequences in tissues other than the brain or early embryo, for example, internal genitalia and anterior silk gland but no epidermis or middle silk gland, which we detected using RT-PCR (see Figure 4 and Supplementary Figure 4). BmorPBP1 and BmorGOBP1 clones are also found in the anterior silk gland of the wild silkmoth *Bombyx mandarina* (A_BomaASGc47494 and A_BomaASGc16510). Therefore, although it should be remembered that the presence of RT-PCR products or even intact mRNA sequences in these tissues does not necessarily imply the presence of functional proteins, the activation of response control elements and detection of transcripts imply PBP/GOBP protein synthesis in tissues as diverse as brain, antennae, legs, gut, epidermis, and silk gland in the silkworm moth *B. mori* (see Figure 4 and Supplementary Figure 4, Supplementary Table 1). High expression levels during whole insect development from the egg (embryo) to late instar larvae, pupae, and adults in a high number of tissues from the brain to silk gland strongly suggest for PBPs and GOBPs some alternate functions to pheromone/odor detection.

The non-antennal specific expression of PBP1, PBP2, GOBP1, and GOBP2 across a number of “non-sensory” tissues raises questions regarding the assigned olfactory role of these proteins (Supplementary Figure 5). Because our data indicate a broader expression profile for the PBP/GOBP clade (Supplementary Figure 5), we posit a new hypothesis in which OBPs are pleiotropic carrier proteins that function in diverse physiological processes, such as CNS function, development, metabolism, and immunity.

Interestingly, high-throughput RNA sequencing (RNA-Seq) of larval transcriptomes in the silkworm challenged by the fungus *Beauveria bassiana* failed to identify PBPs and GOBPs during the early response to infection (Hou et al., 2014). This could indicate that different methods have different sensitivity analyses and/or that PBPs and GOBPs are expressed under specific physiological conditions, i.e., downregulated for fungal infection. Therefore, we checked for evidence of variations in *BmorPBP1, BmorPBP2, BmorGOBP1*, and *BmorGOBP2* expressions in response to chemical stress/abamectin exposure using specific qRT-PCR and following Xuan et al. (2015). There could be multi levels of insecticide resistance in insects, enrolling more genes than the one typically involved in immunological responses under chemical stress conditions, including not only cytochrome P450s, carboxylesterases, and acetylcholinesterase but also small soluble binding proteins, such as “CSPs” (Mamidala et al., 2012; David et al., 2014; Xuan et al., 2015; Einhorn and Imler, 2019). Like THP12 (12 kDa *Tenebrio* hemolymph protein precursor), numerous OBPs are described as immune proteins in the insect hemolymph involved with microbial toxin infection (Graham et al., 2003; Levy et al., 2004; Song et al., 2006; Contreras et al., 2013; Behrens et al., 2014; Hou et al., 2014; Einhorn and Imler, 2019). In particular, antennal binding protein (ABP)-7 is known to be upregulated in the plasma of silkworm larvae in an innate immune response to bacterial stress/*Bacillus* exposure (Song et al., 2006). More recently, symbiotic bacteria, such as the obligate mutualist *Wigglesworthia*, have been shown to induce OBP synthesis in insect gut to maintain hematopoiesis and regulate symbiont-mediated immunological pathways, such as melanotic response in tsetse flies (Benoit et al., 2017; Rihani et al., 2021). These studies are in line with our results from using the pesticide active substance abamectin on several sensory and metabolic tissues from *B. mori* (see Figure 5 and Supplementary Figure 5). Abamectin (avermectins B1a/B1b) is not used in the rearing of the silkworm. It has been chosen because of its insecticide activity in the context of targeting muscles and neurons, i.e., various internal tissues [potentiating gamma-aminobutyric acid (GABA) effects on gated chloride channels], and because it is closely related to macrocyclic lactones produced by soil bacteria. *B. mori* males were chosen because we challenged the moth PBP/GOBP function exclusively tuned to sex pheromone responsiveness (see Figures 1–4 and Supplementary Figures 1–4, Supplementary Table 1). Here, we demonstrate the induction of *PBP/GOBP* genes by the natural product abamectin (see Figure 5), which could be a very important result for insect pest control. If the OBPs are knocked out, it could be that the moths, pupae, or larvae become much more susceptible to abamectin, which is used as an insecticide. This is a new line of research for OBP knockout, insect physiology, and potential application for pest control.

Very interestingly, the increased expression of genes in the moth *PBP/GOBP* clade, in response to abamectin exposure, is found to be tissue-specific, as found for “*CSPs*” and *cytochrome P450* “*CYPs”* (see Figure 5; Xuan et al., 2015). Similar to *CSPs* and *CYPs*, the increased expression of *PBP* and *GOBP* genes after insecticide exposure mainly occurred in tissues with high metabolic rate, such as the gut, thorax (prothoracic glands), and fat body (see Figure 5 and Supplementary Figure 5; Xuan et al., 2015). We also find that the *BmorPBP1* and *BmorGOBP2* genes have significant hits (85–87%) in the EST database from the midgut of another bombycid species, *Trilocha varians* (TrvaMGcomp3213 and TrvaMGcomp652766; silkbase.ab.a.u-tokyo.ac.jp), which is very congruent with our qRT-PCR data. The immunoblot comparisons for the same tissues are not always compatible with those revealed by the analysis of the RNA transcripts in PCR or Expressed Sequence Tags. Transcript levels by themselves are not sufficient to predict protein levels because of various regulatory cellular processes, i.e., RNA and protein synthesis and turnover. There could be a delay (> 6 h) for high effects of abamectin at the protein level (see Figure 5A), which could be detected further by LC/MS/MS. This would, perhaps, reveal additional OBPs induced by the insecticide. Here, the sequences of immunoreactive bands were not confirmed by LC/MS/MS analysis, but the expression of PBP/GOBP outside the olfactory system was confirmed by molecular biology analysis. We did not use immunoblot and protein data as a tool to measure the PBP/GOBP protein copy number per tissue. We checked for the presence of proteins in the PBP/GOBP clade outside the antennal olfactory system in response to insecticide exposure. We performed quantitative real-time PCR to assess relative RNA copy numbers per tissue, therefore showing an interesting link between *OBP* expression and metabolic tissues, such as the gut, thorax (ecdysteroids), and fat body (see Figure 5B). Both qRT-PCR and immunoblot experiments show PBP/GOBP expression outside the olfactory system, which is not a so surprising result. BmorPBP1 rather enhances the sensitivity, but not the selectivity, of pheromone detection (Shiota et al., 2018). Robust olfactory responses are observed in the absence of OBPs (Xiao et al., 2019). The pheromone specificity of PBPs has been revisited in the giant silk moth *A. polyphemus* (Saturniid) and the cabbage moth *Mamestra brassicae* (Noctuid). Using a fluorescence binding assay and several fatty acid ligand molecules brings into doubt the first overwhelmingly held belief that PBP is only tuned to a specific cognate sex pheromone compound (Campanacci et al., 2001; Lautenschlager et al., 2007). Then, an increasing number of *OBP* genes are reported to be expressed in many fluids and tissues as various as tarsi, legs, hemocytes, salivary gland, pheromone gland, prothoracic gland, fat body, gut, epidermis, testis, and wings in a lot of insect species (Li et al., 2008; Okamoto et al., 2008; Xuan et al., 2014; Song et al., 2016; Benoit et al., 2017; Sun et al., 2018; Einhorn and Imler, 2019; Picimbon, 2019; Zhang et al., 2020; Rihani et al., 2021). Some OBP proteins even help in the hemolymph transport of juvenile hormone or JH (Kim et al., 2017). Not only the developmental profiling but also the response to abamectin exposure, fat body, gut, and thorax expression for *PBP/GOBP* clade, and expression co-fluctuation with metabolic and endocrine genes (see Figures 2–5 and Supplementary Figures 1–5) argue for a compact alternative to the traditional olfaction expression, a function far from any olfactory component or semiochemical substance for this clade of moth protein genes or even for the whole insect OBP protein gene family.

Prior to initiating behavioral studies with mutants, we sought to more extensively assess the role of insecticide exposure in *PBP/GOBP* expression. A first hypothesis would be that abamectin exposure leads to induction because PBP/GOBP binds this compound in an innate immune response to chemical insecticide. This is rather very unlikely because the large size of abamectin (C_95_H_142_O_28_) is not appropriate to accommodate the OBP binding site (Sandler et al., 2000; Zhou et al., 2009). In addition, this would not explain the induction of *PBPs* and *GOBPs* in the absence of abamectin at many different stages of moth development. There could be multiple sources of induction of *OBP* gene expressions, such as lipid and hormonal signaling pathways that are activated during development and/or abamectin insecticide stress (see Figures 4, 5 and Supplementary Figure 5). We leaped from gene expression profiling of tissues and developmental stages to a more functional interpretation of PBP and GOBP using the molecular docking modeling approach to assess possible candidates for “non-chemosensory” ligands. Besides insecticides, hormones, and fatty acids, vitamins were chosen, because insects need high content of these nutrients for multiple physiological functions, from cell growth to immune response (Krivosheina, 2008; Salem et al., 2014; Basset et al., 2017). Vitamin K1 was chosen because its structure (and conformation) is very similar to that of Bombykol. Accordingly, our molecular docking results in Linux, PyMOL, and Autodock/Vina suggest a specific binding site and critical amino acid residues (Ser56, Phe12, and Phe118) for binding of a non-semiochemical ligand, such as vitamin phylloquinone K1 in BmorPBP1 (see Figures 6, 7 and Supplementary Figures 6–8, Tables 1, 2 and Supplementary Tables 2, 3, Zenodo dataset). The biological relevance of *in silico* binding of vitamin K and other compounds needs to be investigated further. Just because it can bind (< 4 Å distance between K1 and PBP1 binding site, same configuration with bombykol, U-shape, and the same critical residues in the protein core, Ser56, Phe12, and Phe118 to anchor the ligand, interaction with vitamin > interaction with bombykol, ΔG: −11.5 vs. −7.4 Kcal/mol) does not necessarily mean it does bind and trigger specific physiological responses. However, the structural data presented here (see Figures 6, 7 and Supplementary Figures 6–8, Tables 1, 2 and Supplementary Tables 2, 3, Zenodo dataset) strongly agree with the broad expression of *PBPs* and *GOBPs* in both time and space, expression in eggs, embryo, and aging moths, and expression under insecticide stress, in the physiological data also presented here (see Figures 1–5 and Supplementary Figures 1–5, Supplementary Table 1). The structure and expression results of OBPs in this study compare with previous studies where only structural and binding data were reported without a link to physiology. Our study was more rigorous in examining spatio/temporal expression patterns for OBP/PBP/GOBP-encoding genes. Although the new hypothesis needs to be ultimately proved by additional *in vitro* binding and X-ray studies, our ontogeny, protein/gene expression, and docking data help us to propose that OBPs, such as PBPs and GOBPs, retain a function tuned to “non-semiochemical” ligands, which will remain to be found addressing specifically transport and binding properties of micronutrients and vitamins rather than pheromones and general odorants in future functional analyses of the moth PBP/GOBP clade.

None of the numerous studies on moths have tested the hypothesis that PBPs and GOBPs are regulatory molecules for the binding of non-semiochemicals. This also applies to all various functional analyses and binding studies conducted on OBPs. Based on our extensive physiological study on the silkworm moth *B. mori*, PBP/GOBP in metabolic processes becomes a strong research hypothesis. Like PBP, GOBP is detected in the brain and the earliest stage during insect development, i.e., early embryo, perhaps suggesting a function in neuroplasticity and/or neurogenesis for these proteins. *PBP/GOBP* expression is also detected in the silk gland and gut of wild silk moth species (*B. mandarina* and *T. varians*). Therefore, the function of PBP/GOBP in the metabolic system (changes in growth and nutrient profiles) does not seem to have been altered by thousands of years of domestication, although comparisons are needed to be made with closely related non-domesticated species of the same genus to make a clear claim. If the *B. mori PBP/GOBP* gene set was knocked out, perhaps by CRISPR-based editing, it would be interesting to see what physiological effects might arise, such as non-responsive males to pheromonal stimulation or, as suggested by our molecular docking, potential vitamin deficiencies.

## Conclusions

We comprehensively analyzed the expression profile of the PBP/GOBP gene set in *B. mori* in response to age, development, tissue specificity, and insecticide exposure. Amazingly enough, 20 years after the structure, this is the first complete survey of the tissue and ontogeny expression of PBP/GOBP in silkworms. Here, we carry out the study around the theme “physiological regulation,” and we investigate this theme using multiple experiments, analyzing this clade at different developmental stages in males and females, and using both molecular and biochemical approaches.

The expression of *Bombyx* PBPs and GOBPs in leg tarsi of aged adult females, as well as in tissues as diverse as an early embryo, brain, and silk gland raises questions regarding the currently accepted paradigm of their functionality that is restricted to male-specific pheromone detection. When the OBPs become extremely well-known, at several levels, but focusing only on interaction with semiochemicals, the induction of PBPs and GOBPs in metabolic tissues in response to abamectin insecticide exposure adds new interest to these two classes of binding proteins that appear to be much more versatile than believed so far. The age-, mating-, development- and tissue-dependent expressions of OBPs have been studied in many insect species; thus, structure in relation with physiology is something expected. The amount of tissues covered, as well as specific physiological conditions (exposure to insecticide), maybe just descriptive, but the description strongly indicates non-olfactory functions for OBPs. The role of PBPs and GOBPs was tuned to olfaction. By docking, we report that vitamins could be selective and potent ligands for PBP/GOBP, which would be in agreement with the PBP/GOBP gene expression profiling revealed here in our study.

## Data Availability Statement

The original contributions presented in the study are included in the article/Supplementary Material, further inquiries can be directed to the corresponding author. Docking data are linked to Zenodo Dataset doi: 10.5281/zenodo.5597323, https://zenodo.org/record/5597323#.YXhGxXxxfIU.

## Author Contributions

JFP conceived and designed the study, dissected all the organs and tissues, prepared the RNA samples, analyzed and interpreted the data, analyzed the complete set of data, and wrote the manuscript. QL reared the silkworms. JFP and GL coordinated the insects and lab works. JFP and XG conceived and carried out behavioral assays, prepared the protein samples from all the tissues, performed the biochemical analyses, and did the biochemical work on infected tissues. XG, NX, GL, HX, and JFP did the molecular biology work (one-step RT-PCR and real-time PCR) and performed real-time PCR on moth tissues infected by abamectin. XG, NX, GL, HX, and JFP ran the insecticide test on *Bombyx* tissues. PA and BO planned and performed the docking experiments and conceived and presented the docking results. PA, BO, and JFP interpreted the docking results. All the authors approved the final version of the manuscript.

## Funding

This study was supported by the Natural Sciences Foundation of Shandong Province (ZR2011CM046) and Overseas Talent, Taishan Scholar (JFP, No. tshw20091015).

## Conflict of Interest

The authors declare that the research was conducted in the absence of any commercial or financial relationships that could be construed as a potential conflict of interest.

## Publisher's Note

All claims expressed in this article are solely those of the authors and do not necessarily represent those of their affiliated organizations, or those of the publisher, the editors and the reviewers. Any product that may be evaluated in this article, or claim that may be made by its manufacturer, is not guaranteed or endorsed by the publisher.

## Supplementary Material

Supplementary Figure 1 Comparative *BmorPBP1, BmorPBP2, BmorGOBP1*, and *BmorGOBP2* gene expression profiles across different age groups in **(A)** male and **(B)** female adult silkworm moths. Focus on OBP ratio and x-fold increase in gene expression from RNA samples (D2–D9) in the same experiment as Figure 2 (Step 1). qRT-PCR results with means (*n* = 9) of *PBP2, GOBP1*, and *GOBP2* compared with *PBP1* used as reference (Step 2: *PBP1* expression = 1). PBP/GOBP expression aging differences between males and females.

Supplementary Figure 2 Detection of BmorPBP1 and BmorGOBP2 proteins in legs of female *Bombyx mori* adults. **(A)** Gel electrophoretic separation of highly concentrated protein samples (5–20 mg/ml) from the fat body (Fb), egg, gut (G), head without antennae (Hd), legs (L), epidermis (Ei), thorax (Th), and wings (Wg) of 4-day-old female adult silkworms. **(B)** Immunoblots of 4-day-old female tissues probed with BmorPBP1, BmorGOBP1, and BmorGOBP2 antibodies. The numbers (kDa) aside from the gels or the blots give the position of protein molecular weight markers (MK). The arrow indicates the position of immunoreactive ~16 kDa proteins in the head and leg samples. The asterisk (^*^) shows PBP/GOBP signals in the leg samples and the PBP1 signal in the head (without antennae) samples.

Supplementary Figure 3 Immunoblots of 11 tissues of 8-day-old adult silkworms probed with BmorPBP1, BmorGOBP1, and BmorGOBP2 antibodies. Co, cocoon; G, gut; Hm, hemolymph; Mc, meconium; PG, pheromone gland; iE, internal (unlaid) eggs; eE, external (laid) eggs; mH, male head (without antennae); fH, female head (without antennae); Cp, cephalic capsule; Ey, compound eyes. The numbers aside the blots give the positions of protein molecular weight markers (kDa). The arrow tip indicates the position of immunoreactive ~16 kDa proteins for PBP1 and GOBP1. The asterisk (^*^) shows PBP/GOBP signals in the male head (without antennae), female head (without antennae), cephalic capsule, compound eyes, and meconium of adult silkworms.

Supplementary Figure 4 Expression profiling of *BmorPBP1, BmorPBP2, BmorGOBP1*, and *BmorGOBP2* genes across 9 distinct tissues in early pupae (E-5) and adult (D1/D9) moths. A, antennae; F, fat body; G, gut; H, head (without antennae); L, legs; P, pheromone gland; Ei, internal envelope (epidermis); T, thorax; W, wings. E-5, 5-days before emergence (no rigid organs); D1, 1-day-old female adult *B. mori*; D9, 9-day-old female adult *B. mori*. The numbers indicate the length in bps of specific RT-PCR products (*CypA, Actin, PBP1, PBP2, GOBP1*, and *GOBP2*). *Actin* and *CypA* (*cyclophilin A*) are used as housekeeping control genes that are expected to express equally across the different tissues. RNA control of tissue samples (1 μg/lane) is shown below. *PBP/GOBP* gene expression profiles in specific tissues are age-dependent. The arrow tip indicates (1) *PBP1* expression in the antennae, head, legs, and epidermis of D1; (2) *PBP2* expression in the antennae, head, legs, thorax, and wings of E-5, (3) *GOBP1* expression in most of all the tissues of D1; and (4) *GOBP2* expression in the antennae, gut, head, legs, epidermis, and wings of D9.

Supplementary Figure 5 Tissue expression profiling of moth *PBP/GOBP*. *B. mori PBP/GOBP* gene expression profiling during the silkworm development from eggs to adults under **(A)** normal conditions and **(B)** following exposure to abamectin insecticide. Data are from Figures 2–5, Supplementary Figures 1–4, and Supplementary Table 1. Specific gene expression is shown by color code: red (PBP1), blue (PBP2), green (GOBP1), and orange (GOBP2). Upregulation in the expression levels of *PBP/GOBP* genes is indicated by a larger circle. Downregulation is indicated by a triangle oriented down. Ant, antennae; Ep, epidermis; FB, fat body; G, gut; Lg, legs; HPL, hair-pencils; Th, thorax; Wg, wings.

Supplementary Figure 6 Configuration and position of bombykol in protein binding site. **(A)** PBP1. **(B)** GOBP2. In blue: linux (theoretical), in red: X-ray (experimental).

Supplementary Figure 7 A repertoire of “non-sensory” chemical structures tested in docking experiments for preferred orientation and interaction when bound to BmorPBP1 and BmorGOBP2 binding sites. **(A)** Vitamins (A–K3). **(B)** Insecticides (neonicotinoids, organophosphates, and pyrethrins). **(C)** Juvenoids (JH and mimetics). **(D)** Caffeine (alkaloid methylxanthines). **(E)** Esters of carboxylic fatty acids (short chains).

Supplementary Figure 8 Docking simulation of vitamin and insecticide pyrethrin molecules bound to BmorGOBP2. Vitamin K2 (pose 1): motif 1, total_Nb 14, Val de Ene (ΔG) = −11.1 Kcal/mol; Vitamin K2 (pose 96): Motif 1, Total_Nb 24, Val de Ene (ΔG) = −10.8 Kcal/mol; vitamin A (pose 18): Motif 1, Total_Nb 32, Val de Ene (ΔG) = −9.5 Kcal/mol; vitamin A (pose 95): motif 1, total_Nb 4, Val de Ene (ΔG) = −10.2 Kcal/mol; vitamin K1 (pose 40): motif 1, total_Nb 1, Val de Ene (ΔG) = −9.1 Kcal/mol; vitamin K1 (pose 18): motif 6, total_Nb 4, Val de Ene (ΔG) = −7.3 Kcal/mol; ergocalciferol (pose 97): motif 1, total_Nb 0, Val de Ene (ΔG) = −9.5 Kcal/mol; ergocalciferol (pose 47): motif 9, total_Nb 35, Val de Ene (ΔG) = −6.5 Kcal/mol; vitamin E (pose 97): motif 1, total_Nb 0, Val de Ene (ΔG) = −10.6 Kcal/mol; vitamin E (pose 66): motif 7, total_Nb 14, Val de Ene (ΔG) = −6.6 Kcal/mol; pyrethrin II (pose 43): motif 1, total_Nb 0, Val de Ene (ΔG) = −9.9 Kcal/mol; pyrethrin II (pose 0): motif 2, total_Nb 23, Val de Ene (ΔG) = −7.5 Kcal/mol. ΔG shows the relative binding affinity value for vitamin compound to BmorGOBP2 protein. Docking pose shows scored matching or “best-fit” of fragment atoms from vitamin and BmorGOBP2 in a multiple-grid arrangment.

Supplementary Table 1 BLASTn analysis of *B. mori* tissues (strain p50T) in silkbase. Silkbase.ab.a.u-tokyo.ac.jp; Br, brain; EE, early embryo; FB, fat body; IG, internal genitalia; Mg, midgut; ASG, anterior silk gland; MSG, middle silk gland; Ep, epidermis; N4EE, early embryo (strain N4). E value 0.0-7e-28.

Supplementary Table 2 Docking parameters for each “non-semiochemical” ligand in BmorPBP1 binding site (Linux). = indicates the same ligand position compared with the geometry adopted by the optimized Bombykol position. ≠ indicates a different position. Sym: symmetrical position. Results are ranked by best binding energy (ΔG) value calculated using the experimentally observed best conformation for each ligand. Ergocalciferol, vitamins K2, K1, E, A, and riboflavin (vitamin B2) are the best ligands (predicted high binding affinity) for the BmorPBP1 binding site (in a conformation similar or symmetrical to that of Bombykol).

Supplementary Table 3 Docking parameters for each “non-semiochemical” ligand in the BmorGOBP2 binding site (Linux). ≠ indicates a different position compared with the optimized Bombykol position. ^*^ indicates that the statistical difference between the position of vitamin K and the position of bombykol in BmorGOBP2 is low. Results are ranked by best binding energy (ΔG) value calculated using the experimentally observed best conformation for each ligand. Vitamin K is the best ligand (predicted high binding affinity in docking experiment) for the BmorGOBP2 binding site (in a position different than the geometry adopted by bombykol).
