# Supplementary figures and images for: An Expanded Survey of the Moth PBP/GOBP Clade in Bombyx mori: New Insight into Expression and Functional Roles
Source: Front Physiol. 2021 Oct 28;12:712593. doi: 10.3389/fphys.2021.712593 (PMC8582636; doi:10.3389/fphys.2021.712593)

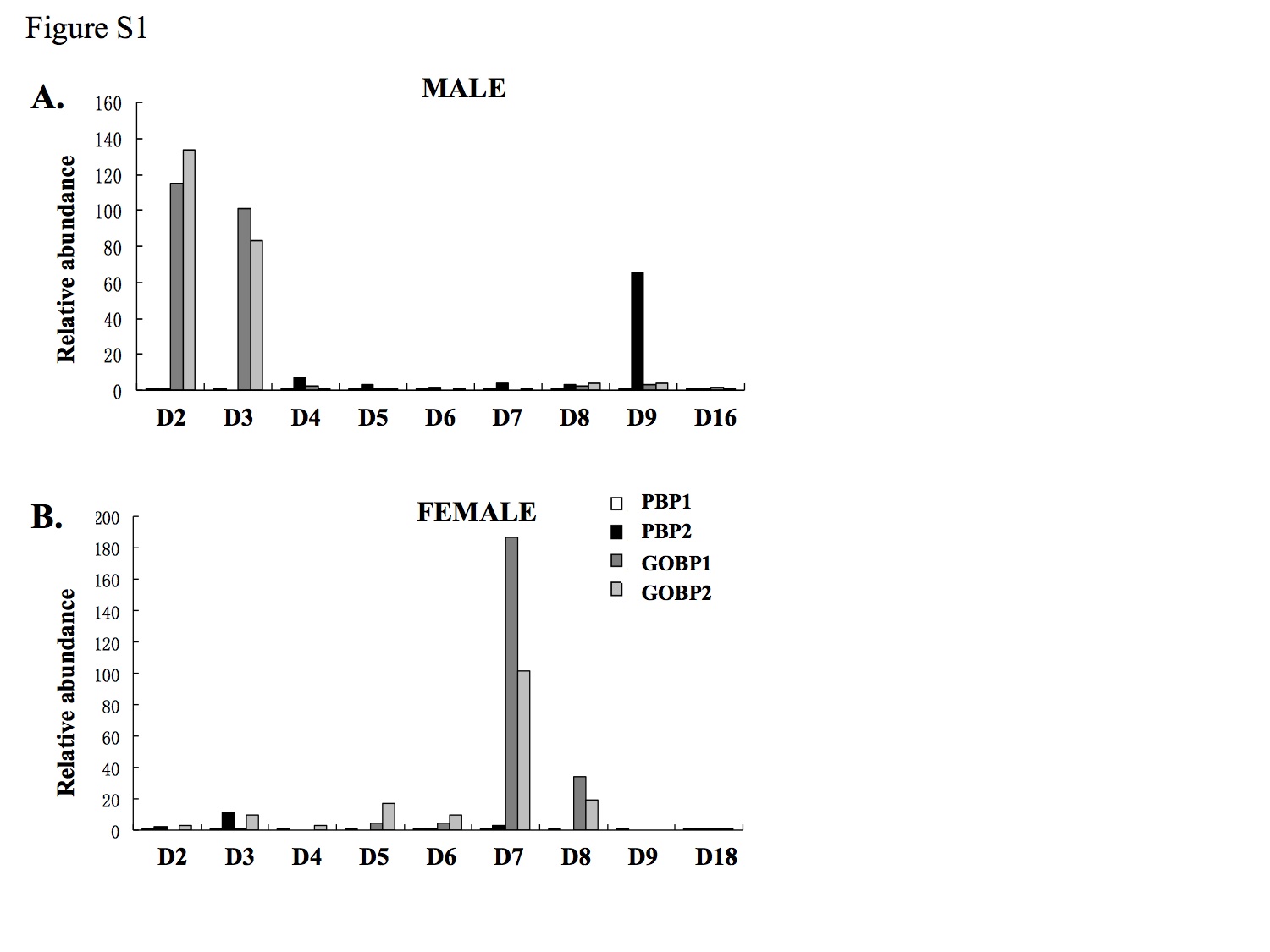

Supplement: Supplementary Figure 1 — Comparative BmorPBP1, BmorPBP2, BmorGOBP1, and BmorGOBP2 gene expression profiles across different age groups in (A) male and (B) female adult silkworm moths. Focus on OBP ratio and x-fold increase in gene expression from RNA samples (D2–D9) in the same experiment as Figure 2 (Step 1). qRT-PCR results with means (n = 9) of PBP2, GOBP1, and GOBP2 compared with PBP1 used as reference (Step 2: PBP1 expression = 1). PBP/GOBP expression aging differences between males and females. [file Data_Sheet_1.zip › Guoetal.FrontPhysiolFINAL2021-SupplMaterials/Guoetal.FrontPhysiol2021-FigureS1.jpg]

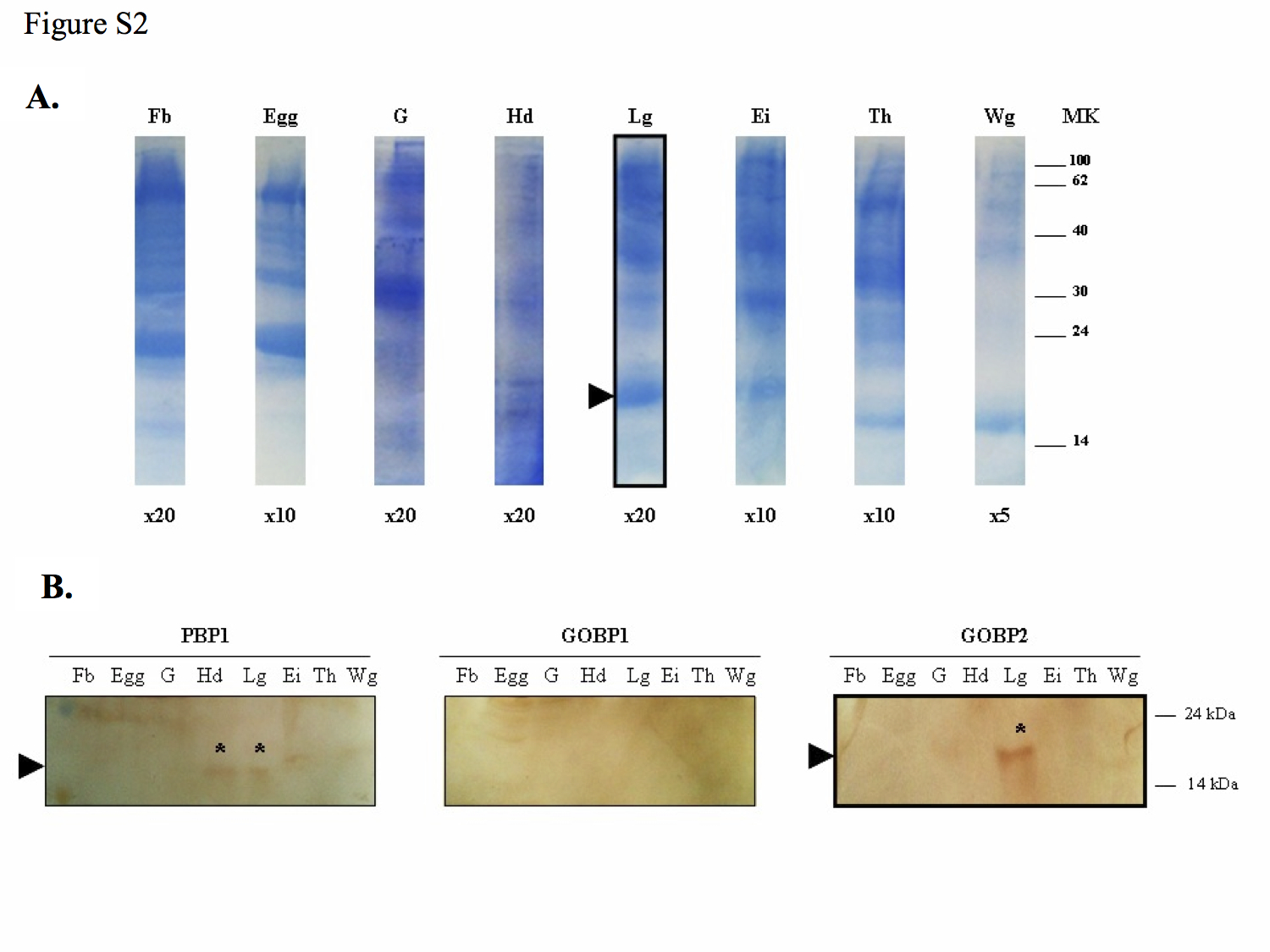

Supplement: Supplementary Figure 1 — Comparative BmorPBP1, BmorPBP2, BmorGOBP1, and BmorGOBP2 gene expression profiles across different age groups in (A) male and (B) female adult silkworm moths. Focus on OBP ratio and x-fold increase in gene expression from RNA samples (D2–D9) in the same experiment as Figure 2 (Step 1). qRT-PCR results with means (n = 9) of PBP2, GOBP1, and GOBP2 compared with PBP1 used as reference (Step 2: PBP1 expression = 1). PBP/GOBP expression aging differences between males and females. [file Data_Sheet_1.zip › Guoetal.FrontPhysiolFINAL2021-SupplMaterials/Guoetal.FrontPhysiol2021-FigureS2.jpg]

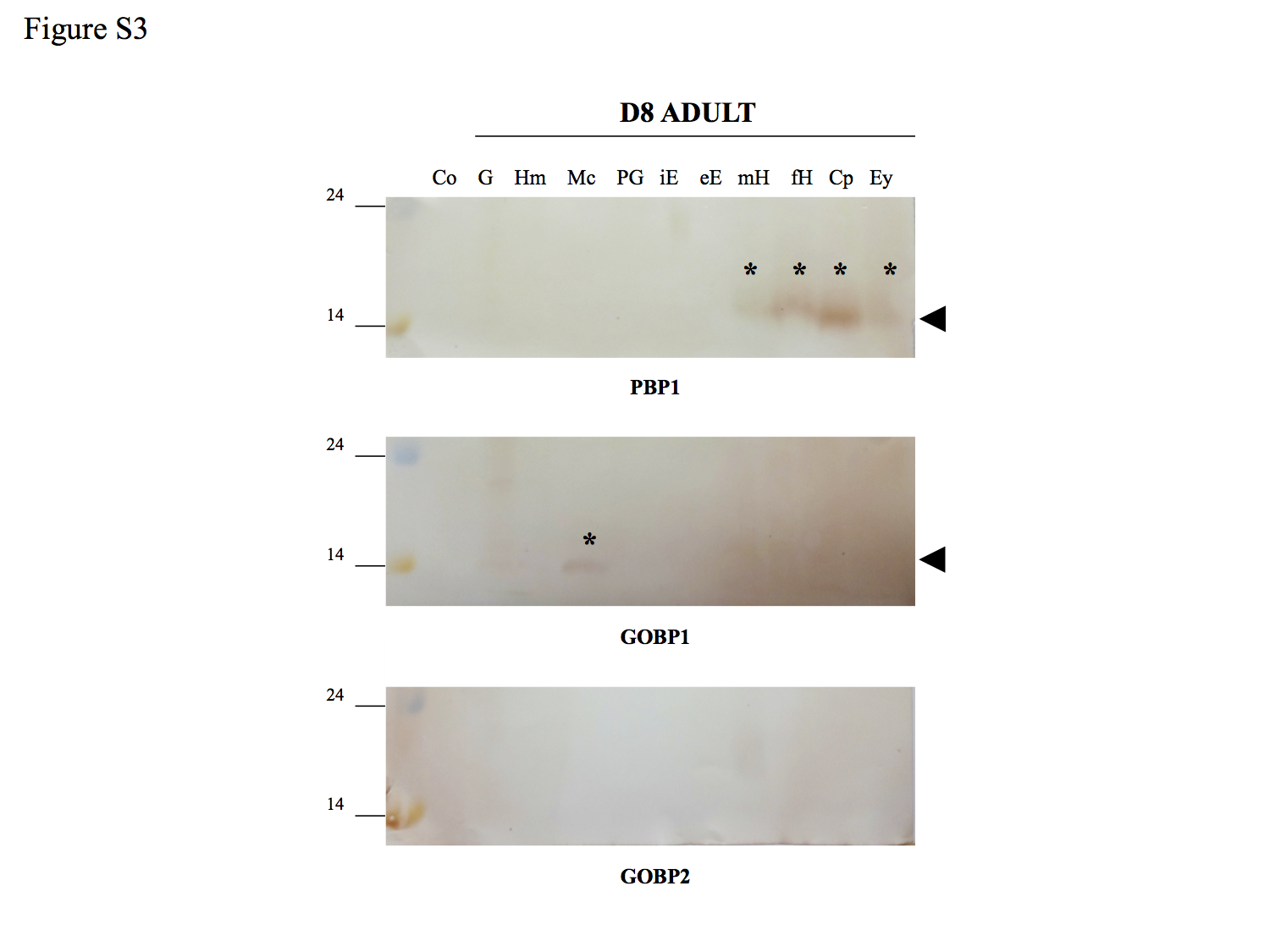

Supplement: Supplementary Figure 1 — Comparative BmorPBP1, BmorPBP2, BmorGOBP1, and BmorGOBP2 gene expression profiles across different age groups in (A) male and (B) female adult silkworm moths. Focus on OBP ratio and x-fold increase in gene expression from RNA samples (D2–D9) in the same experiment as Figure 2 (Step 1). qRT-PCR results with means (n = 9) of PBP2, GOBP1, and GOBP2 compared with PBP1 used as reference (Step 2: PBP1 expression = 1). PBP/GOBP expression aging differences between males and females. [file Data_Sheet_1.zip › Guoetal.FrontPhysiolFINAL2021-SupplMaterials/Guoetal.FrontPhysiol2021-FigureS3.jpg]

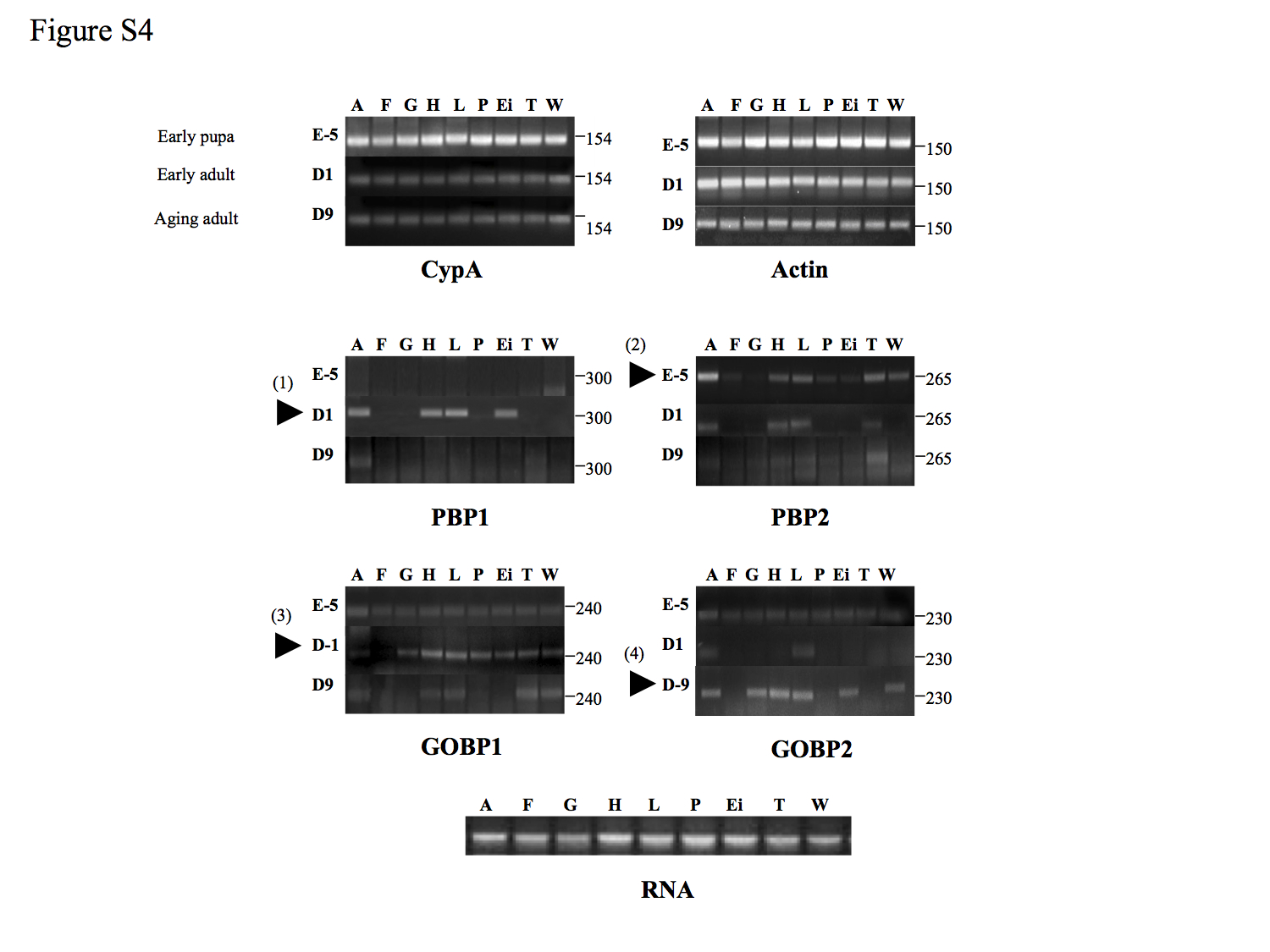

Supplement: Supplementary Figure 1 — Comparative BmorPBP1, BmorPBP2, BmorGOBP1, and BmorGOBP2 gene expression profiles across different age groups in (A) male and (B) female adult silkworm moths. Focus on OBP ratio and x-fold increase in gene expression from RNA samples (D2–D9) in the same experiment as Figure 2 (Step 1). qRT-PCR results with means (n = 9) of PBP2, GOBP1, and GOBP2 compared with PBP1 used as reference (Step 2: PBP1 expression = 1). PBP/GOBP expression aging differences between males and females. [file Data_Sheet_1.zip › Guoetal.FrontPhysiolFINAL2021-SupplMaterials/Guoetal.FrontPhysiol2021-FigureS4.jpg]

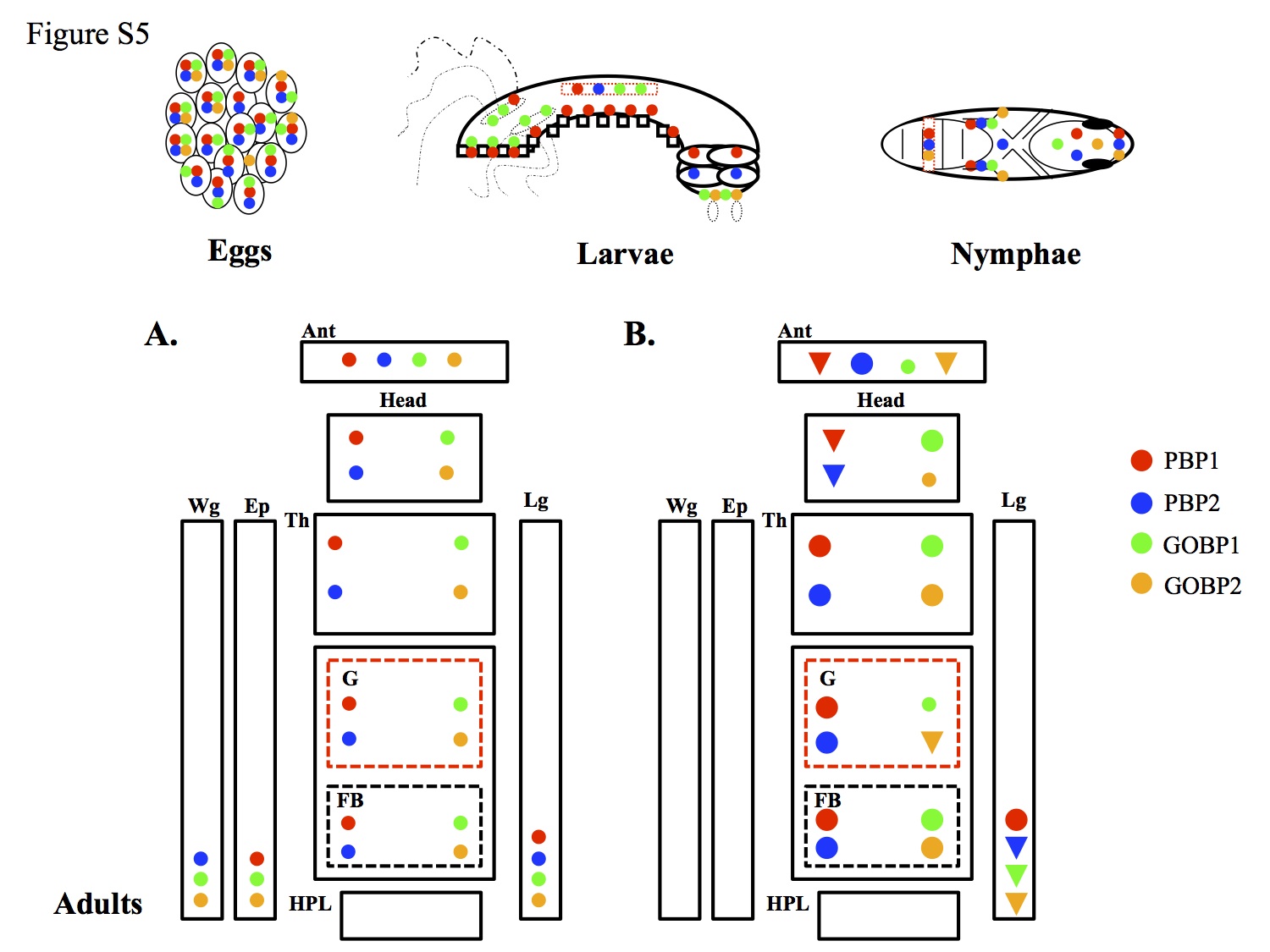

Supplement: Supplementary Figure 1 — Comparative BmorPBP1, BmorPBP2, BmorGOBP1, and BmorGOBP2 gene expression profiles across different age groups in (A) male and (B) female adult silkworm moths. Focus on OBP ratio and x-fold increase in gene expression from RNA samples (D2–D9) in the same experiment as Figure 2 (Step 1). qRT-PCR results with means (n = 9) of PBP2, GOBP1, and GOBP2 compared with PBP1 used as reference (Step 2: PBP1 expression = 1). PBP/GOBP expression aging differences between males and females. [file Data_Sheet_1.zip › Guoetal.FrontPhysiolFINAL2021-SupplMaterials/Guoetal.FrontPhysiol2021-FigureS5.jpg]

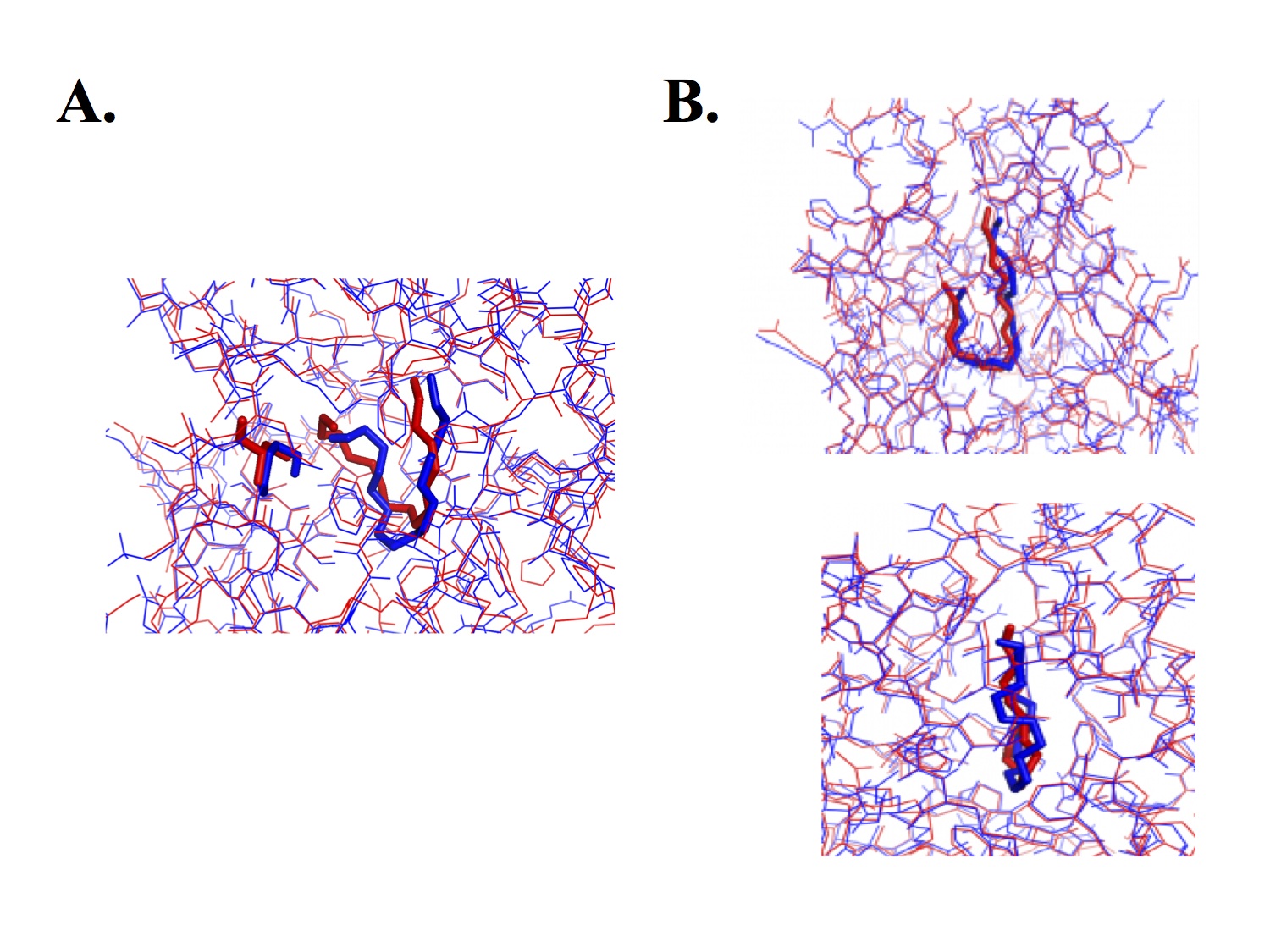

Supplement: Supplementary Figure 1 — Comparative BmorPBP1, BmorPBP2, BmorGOBP1, and BmorGOBP2 gene expression profiles across different age groups in (A) male and (B) female adult silkworm moths. Focus on OBP ratio and x-fold increase in gene expression from RNA samples (D2–D9) in the same experiment as Figure 2 (Step 1). qRT-PCR results with means (n = 9) of PBP2, GOBP1, and GOBP2 compared with PBP1 used as reference (Step 2: PBP1 expression = 1). PBP/GOBP expression aging differences between males and females. [file Data_Sheet_1.zip › Guoetal.FrontPhysiolFINAL2021-SupplMaterials/Guoetal.FrontPhysiol2021-FigureS6.jpg]

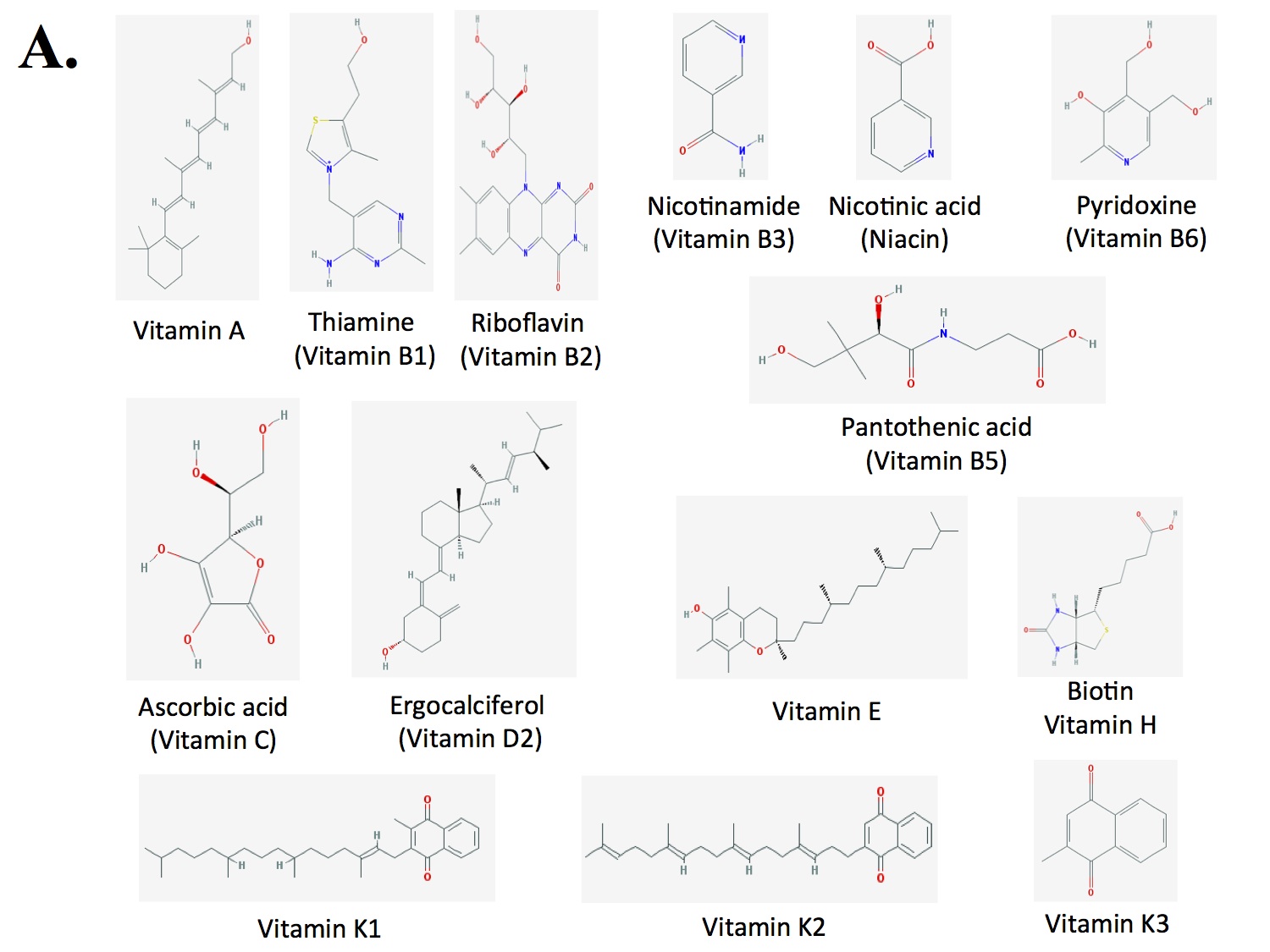

Supplement: Supplementary Figure 1 — Comparative BmorPBP1, BmorPBP2, BmorGOBP1, and BmorGOBP2 gene expression profiles across different age groups in (A) male and (B) female adult silkworm moths. Focus on OBP ratio and x-fold increase in gene expression from RNA samples (D2–D9) in the same experiment as Figure 2 (Step 1). qRT-PCR results with means (n = 9) of PBP2, GOBP1, and GOBP2 compared with PBP1 used as reference (Step 2: PBP1 expression = 1). PBP/GOBP expression aging differences between males and females. [file Data_Sheet_1.zip › Guoetal.FrontPhysiolFINAL2021-SupplMaterials/Guoetal.FrontPhysiol2021-FigureS7A.jpg]

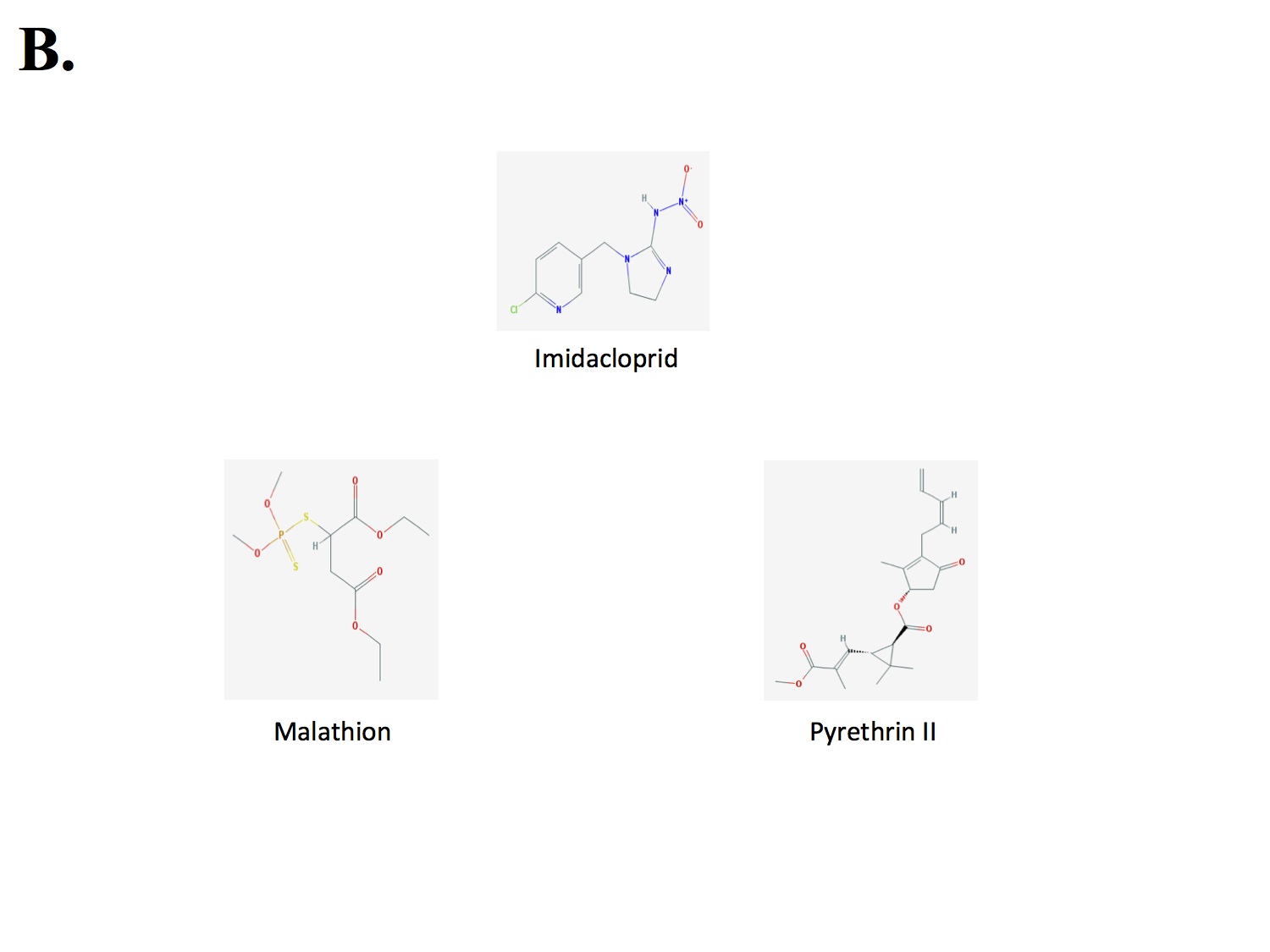

Supplement: Supplementary Figure 1 — Comparative BmorPBP1, BmorPBP2, BmorGOBP1, and BmorGOBP2 gene expression profiles across different age groups in (A) male and (B) female adult silkworm moths. Focus on OBP ratio and x-fold increase in gene expression from RNA samples (D2–D9) in the same experiment as Figure 2 (Step 1). qRT-PCR results with means (n = 9) of PBP2, GOBP1, and GOBP2 compared with PBP1 used as reference (Step 2: PBP1 expression = 1). PBP/GOBP expression aging differences between males and females. [file Data_Sheet_1.zip › Guoetal.FrontPhysiolFINAL2021-SupplMaterials/Guoetal.FrontPhysiol2021-FigureS7B.jpg]

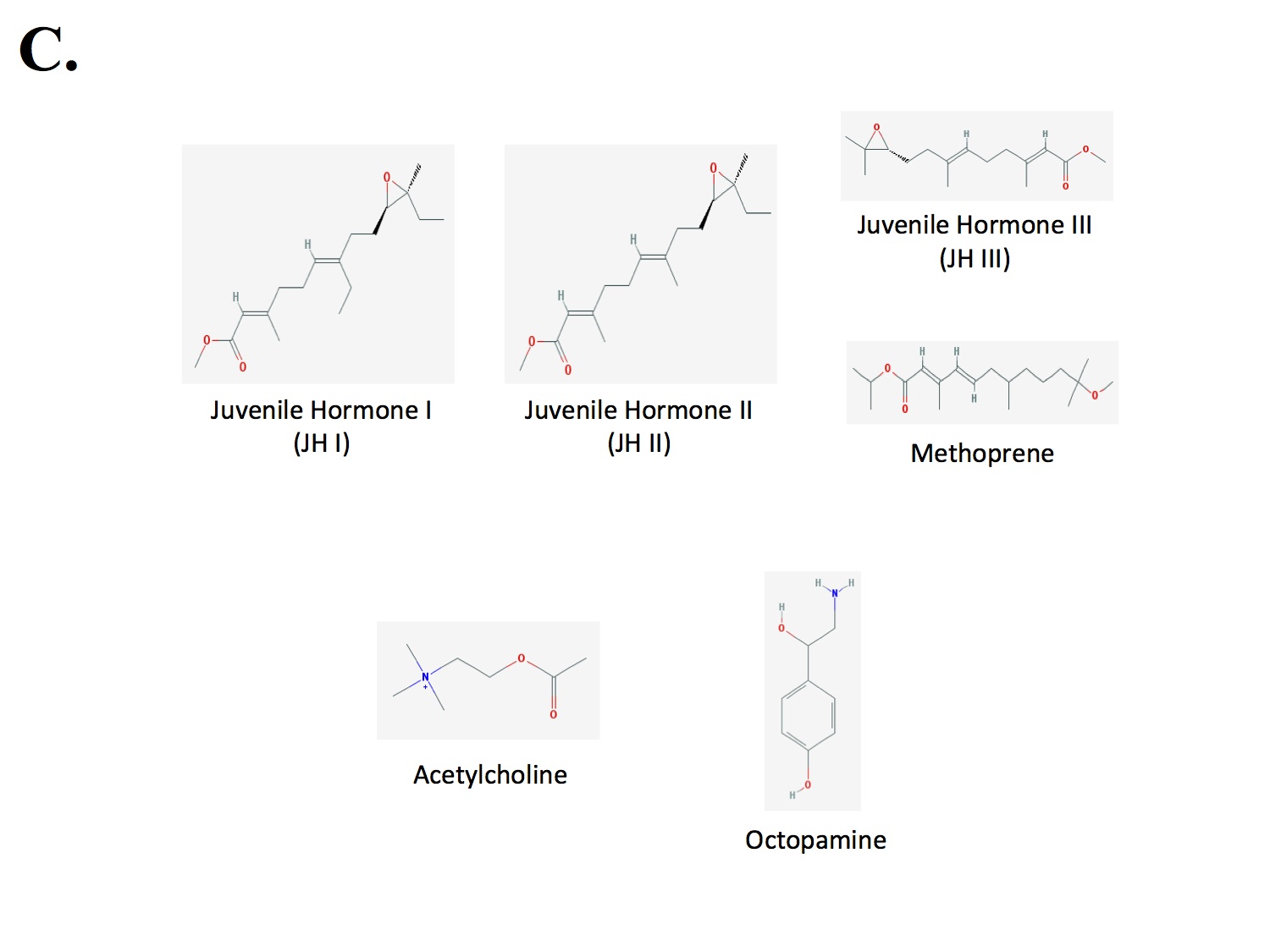

Supplement: Supplementary Figure 1 — Comparative BmorPBP1, BmorPBP2, BmorGOBP1, and BmorGOBP2 gene expression profiles across different age groups in (A) male and (B) female adult silkworm moths. Focus on OBP ratio and x-fold increase in gene expression from RNA samples (D2–D9) in the same experiment as Figure 2 (Step 1). qRT-PCR results with means (n = 9) of PBP2, GOBP1, and GOBP2 compared with PBP1 used as reference (Step 2: PBP1 expression = 1). PBP/GOBP expression aging differences between males and females. [file Data_Sheet_1.zip › Guoetal.FrontPhysiolFINAL2021-SupplMaterials/Guoetal.FrontPhysiol2021-FigureS7C.jpg]

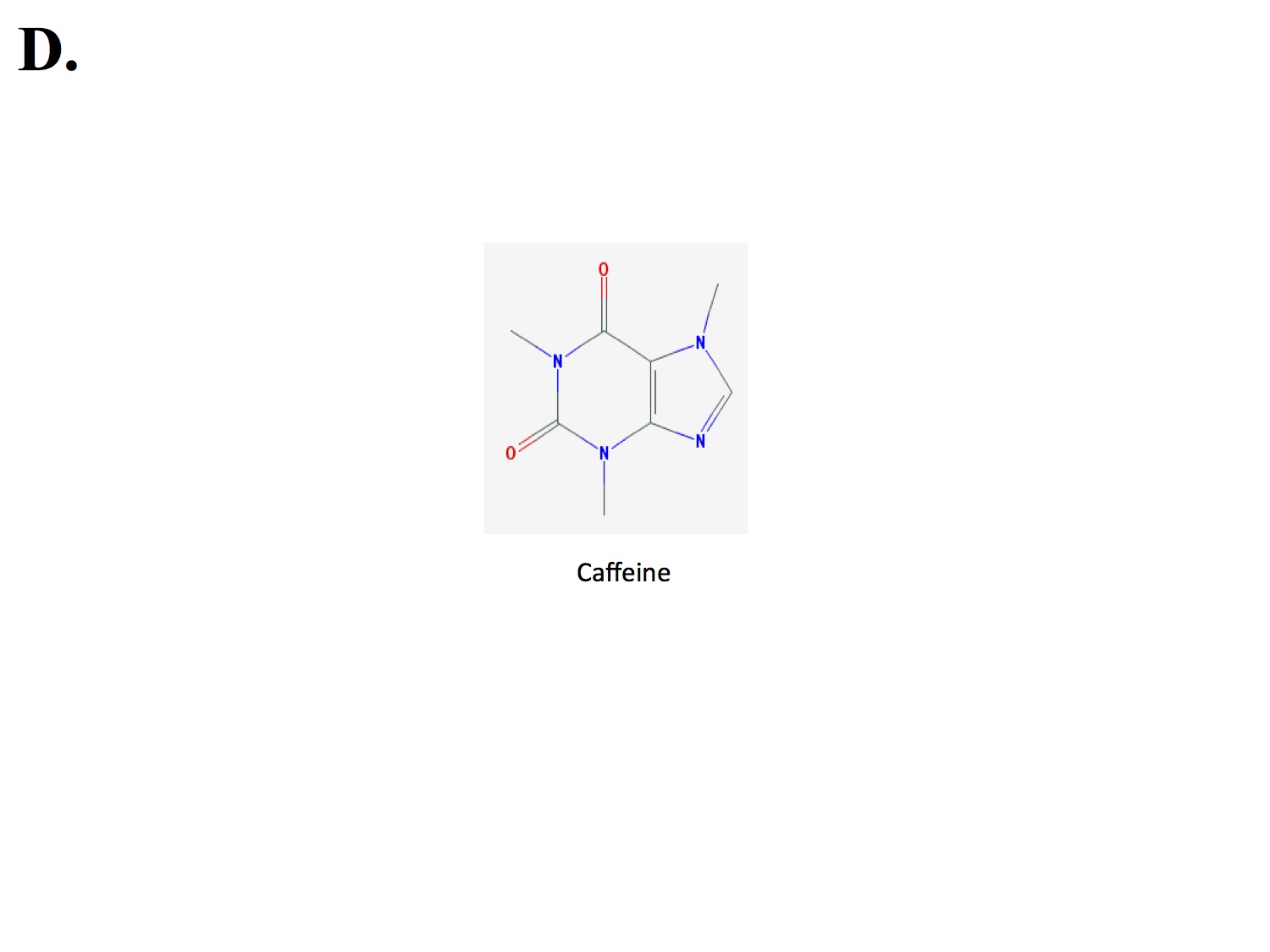

Supplement: Supplementary Figure 1 — Comparative BmorPBP1, BmorPBP2, BmorGOBP1, and BmorGOBP2 gene expression profiles across different age groups in (A) male and (B) female adult silkworm moths. Focus on OBP ratio and x-fold increase in gene expression from RNA samples (D2–D9) in the same experiment as Figure 2 (Step 1). qRT-PCR results with means (n = 9) of PBP2, GOBP1, and GOBP2 compared with PBP1 used as reference (Step 2: PBP1 expression = 1). PBP/GOBP expression aging differences between males and females. [file Data_Sheet_1.zip › Guoetal.FrontPhysiolFINAL2021-SupplMaterials/Guoetal.FrontPhysiol2021-FigureS7D.jpg]

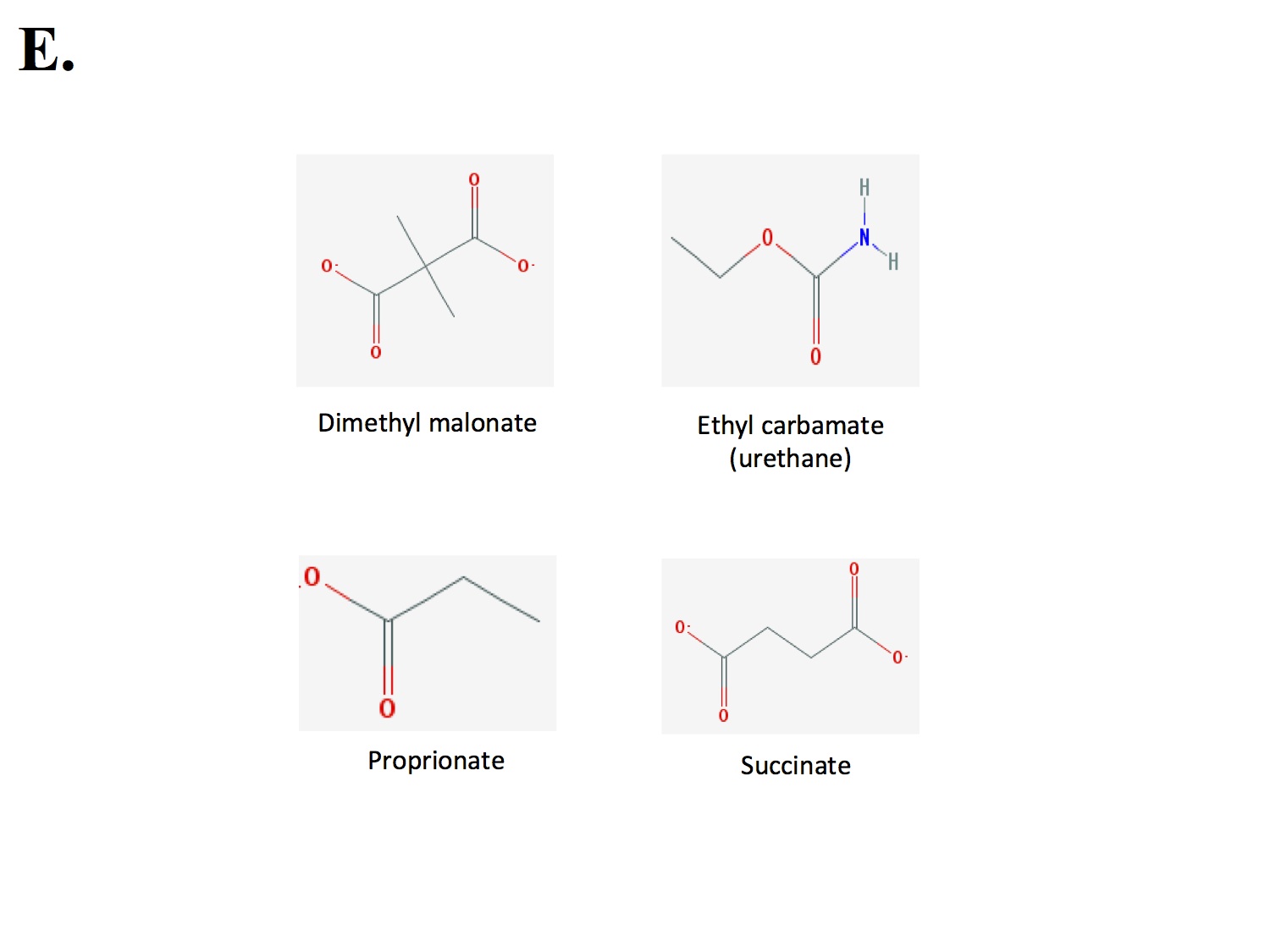

Supplement: Supplementary Figure 1 — Comparative BmorPBP1, BmorPBP2, BmorGOBP1, and BmorGOBP2 gene expression profiles across different age groups in (A) male and (B) female adult silkworm moths. Focus on OBP ratio and x-fold increase in gene expression from RNA samples (D2–D9) in the same experiment as Figure 2 (Step 1). qRT-PCR results with means (n = 9) of PBP2, GOBP1, and GOBP2 compared with PBP1 used as reference (Step 2: PBP1 expression = 1). PBP/GOBP expression aging differences between males and females. [file Data_Sheet_1.zip › Guoetal.FrontPhysiolFINAL2021-SupplMaterials/Guoetal.FrontPhysiol2021-FigureS7E .jpg]

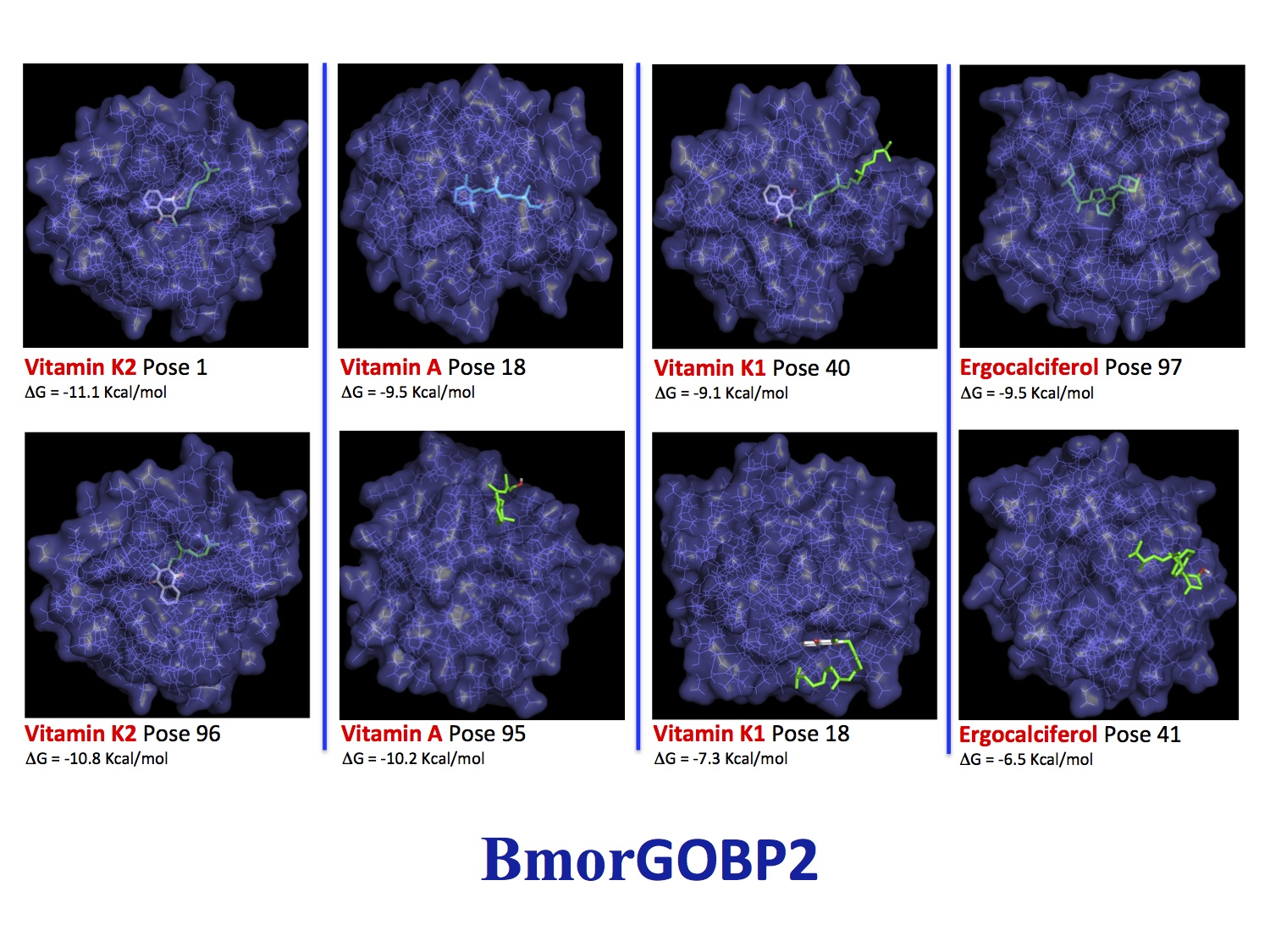

Supplement: Supplementary Figure 1 — Comparative BmorPBP1, BmorPBP2, BmorGOBP1, and BmorGOBP2 gene expression profiles across different age groups in (A) male and (B) female adult silkworm moths. Focus on OBP ratio and x-fold increase in gene expression from RNA samples (D2–D9) in the same experiment as Figure 2 (Step 1). qRT-PCR results with means (n = 9) of PBP2, GOBP1, and GOBP2 compared with PBP1 used as reference (Step 2: PBP1 expression = 1). PBP/GOBP expression aging differences between males and females. [file Data_Sheet_1.zip › Guoetal.FrontPhysiolFINAL2021-SupplMaterials/Guoetal.FrontPhysiol2021-FigureS8.jpg]

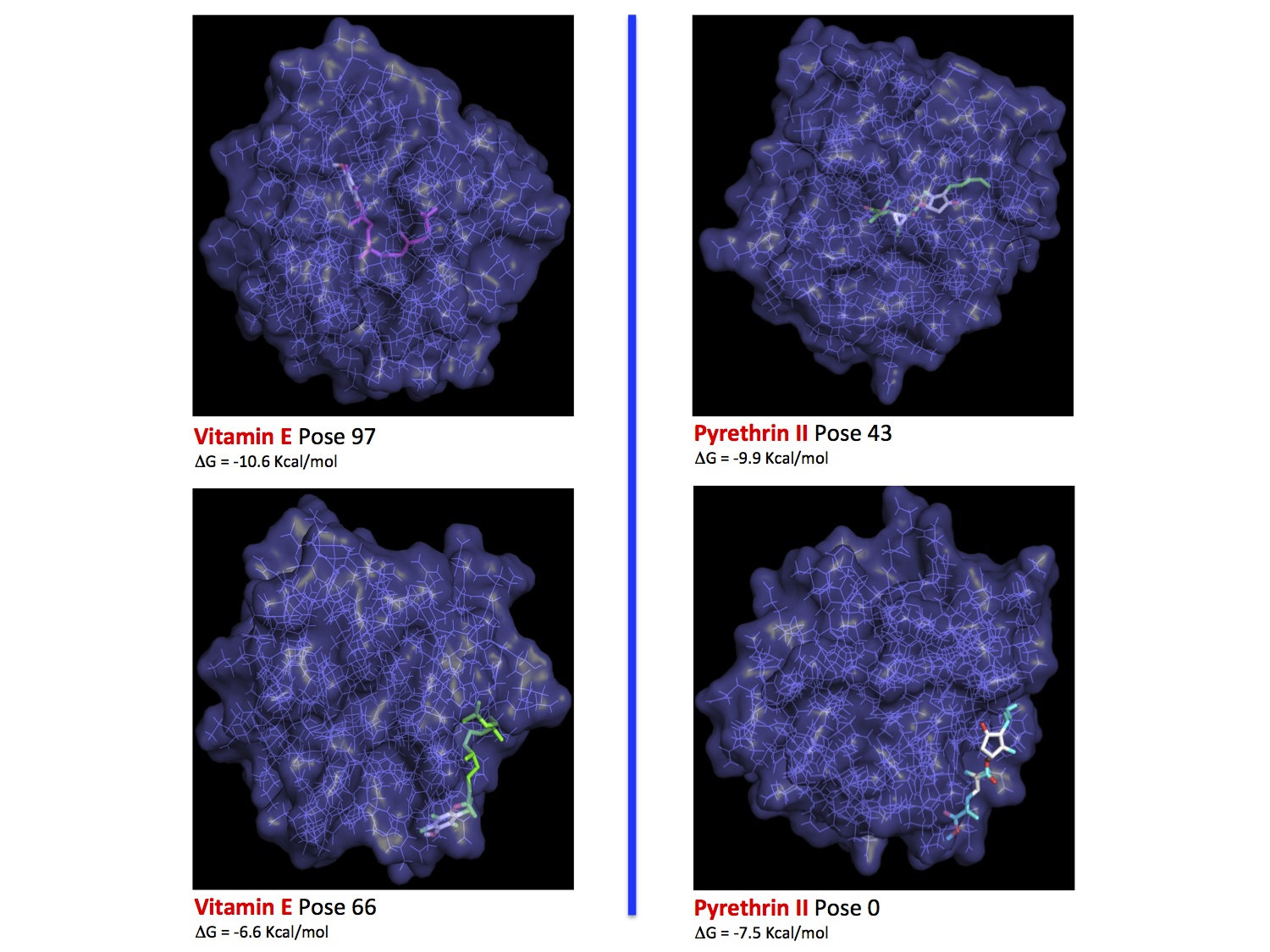

Supplement: Supplementary Figure 1 — Comparative BmorPBP1, BmorPBP2, BmorGOBP1, and BmorGOBP2 gene expression profiles across different age groups in (A) male and (B) female adult silkworm moths. Focus on OBP ratio and x-fold increase in gene expression from RNA samples (D2–D9) in the same experiment as Figure 2 (Step 1). qRT-PCR results with means (n = 9) of PBP2, GOBP1, and GOBP2 compared with PBP1 used as reference (Step 2: PBP1 expression = 1). PBP/GOBP expression aging differences between males and females. [file Data_Sheet_1.zip › Guoetal.FrontPhysiolFINAL2021-SupplMaterials/Guoetal.FrontPhysiol2021-FigureS8continued .jpg]

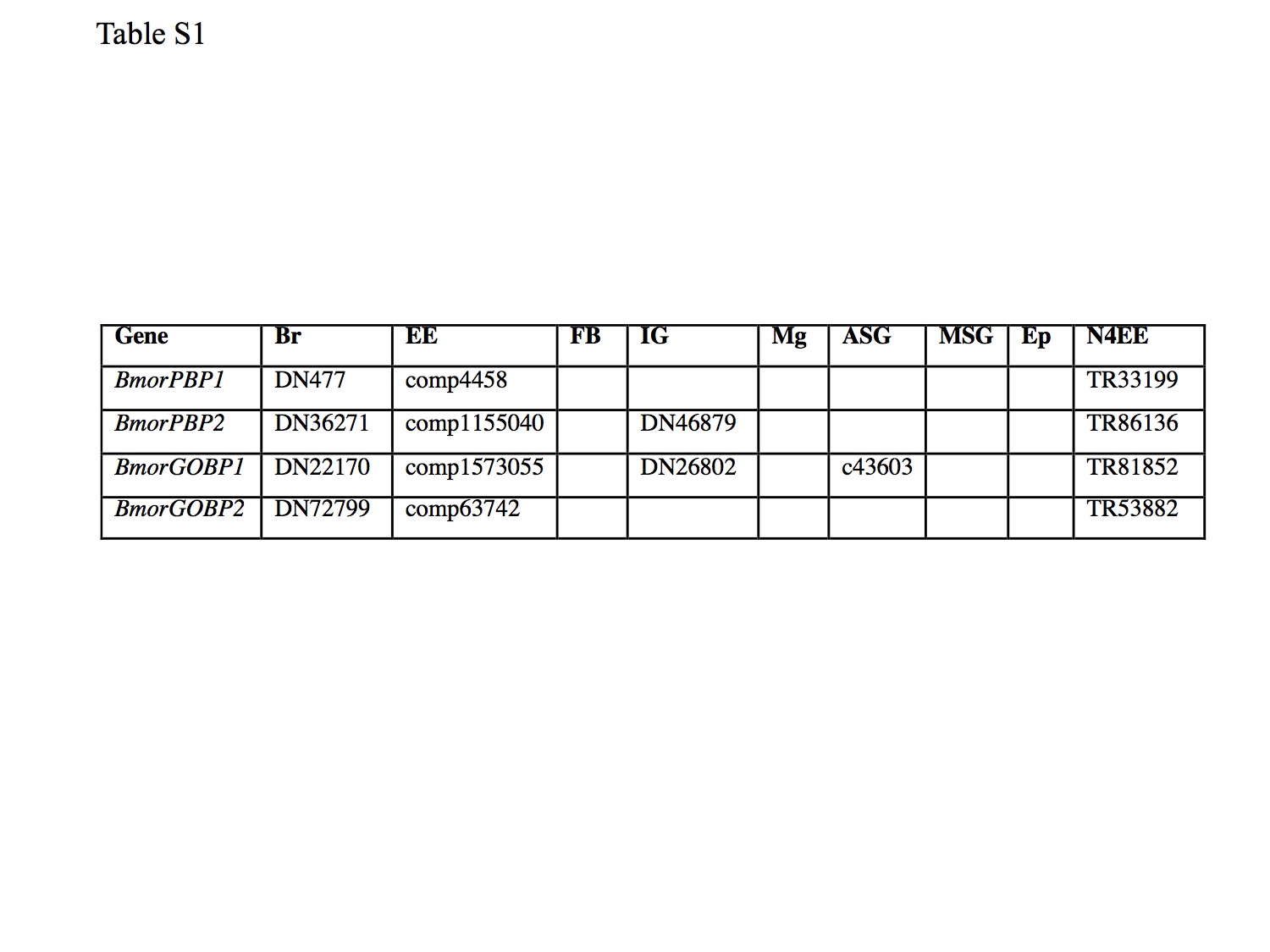

Supplement: Supplementary Figure 1 — Comparative BmorPBP1, BmorPBP2, BmorGOBP1, and BmorGOBP2 gene expression profiles across different age groups in (A) male and (B) female adult silkworm moths. Focus on OBP ratio and x-fold increase in gene expression from RNA samples (D2–D9) in the same experiment as Figure 2 (Step 1). qRT-PCR results with means (n = 9) of PBP2, GOBP1, and GOBP2 compared with PBP1 used as reference (Step 2: PBP1 expression = 1). PBP/GOBP expression aging differences between males and females. [file Data_Sheet_1.zip › Guoetal.FrontPhysiolFINAL2021-SupplMaterials/Guoetal.FrontPhysiol2021-TableS1.jpg]

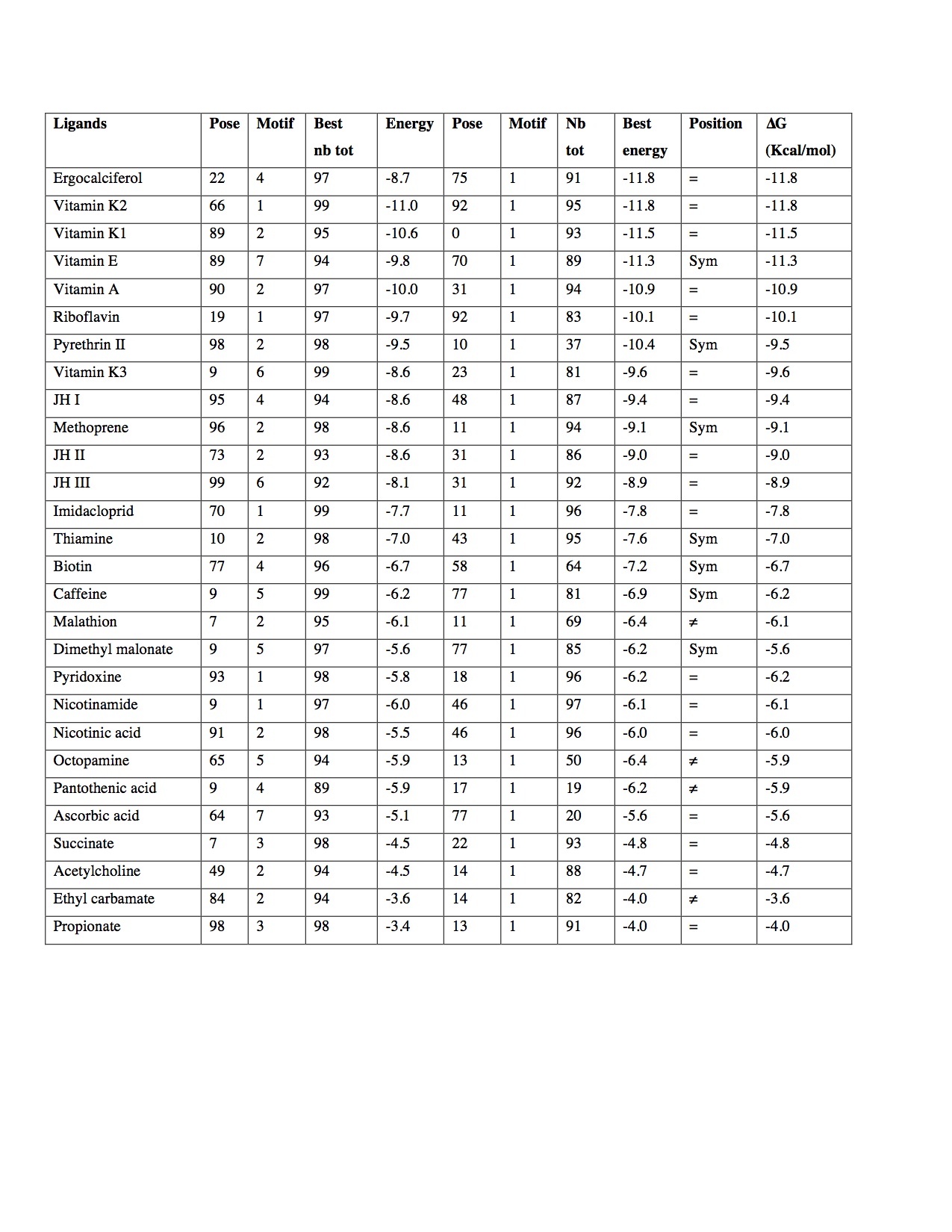

Supplement: Supplementary Figure 1 — Comparative BmorPBP1, BmorPBP2, BmorGOBP1, and BmorGOBP2 gene expression profiles across different age groups in (A) male and (B) female adult silkworm moths. Focus on OBP ratio and x-fold increase in gene expression from RNA samples (D2–D9) in the same experiment as Figure 2 (Step 1). qRT-PCR results with means (n = 9) of PBP2, GOBP1, and GOBP2 compared with PBP1 used as reference (Step 2: PBP1 expression = 1). PBP/GOBP expression aging differences between males and females. [file Data_Sheet_1.zip › Guoetal.FrontPhysiolFINAL2021-SupplMaterials/Guoetal.FrontPhysiol2021-TableS2.jpg]

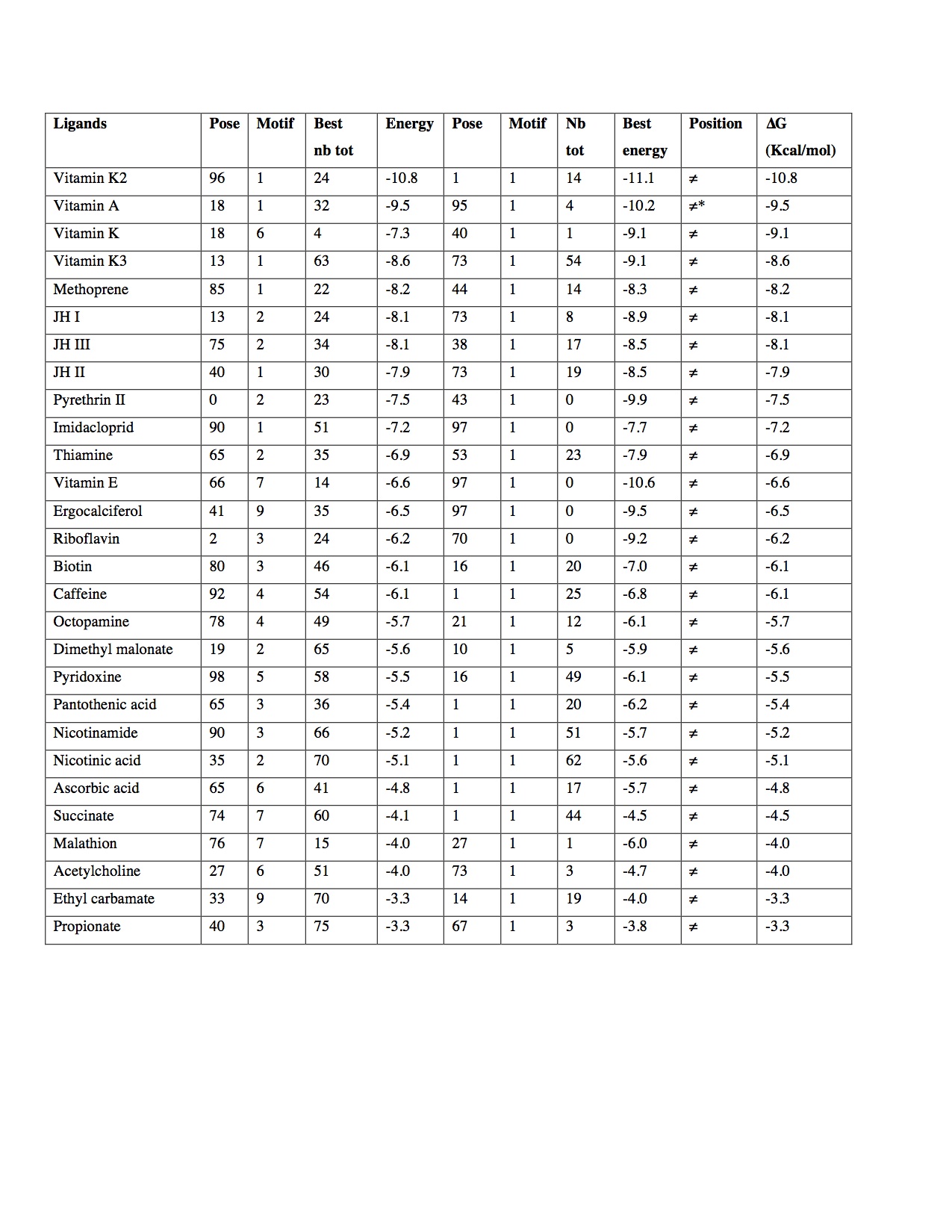

Supplement: Supplementary Figure 1 — Comparative BmorPBP1, BmorPBP2, BmorGOBP1, and BmorGOBP2 gene expression profiles across different age groups in (A) male and (B) female adult silkworm moths. Focus on OBP ratio and x-fold increase in gene expression from RNA samples (D2–D9) in the same experiment as Figure 2 (Step 1). qRT-PCR results with means (n = 9) of PBP2, GOBP1, and GOBP2 compared with PBP1 used as reference (Step 2: PBP1 expression = 1). PBP/GOBP expression aging differences between males and females. [file Data_Sheet_1.zip › Guoetal.FrontPhysiolFINAL2021-SupplMaterials/Guoetal.FrontPhysiol2021-TableS3.jpg]

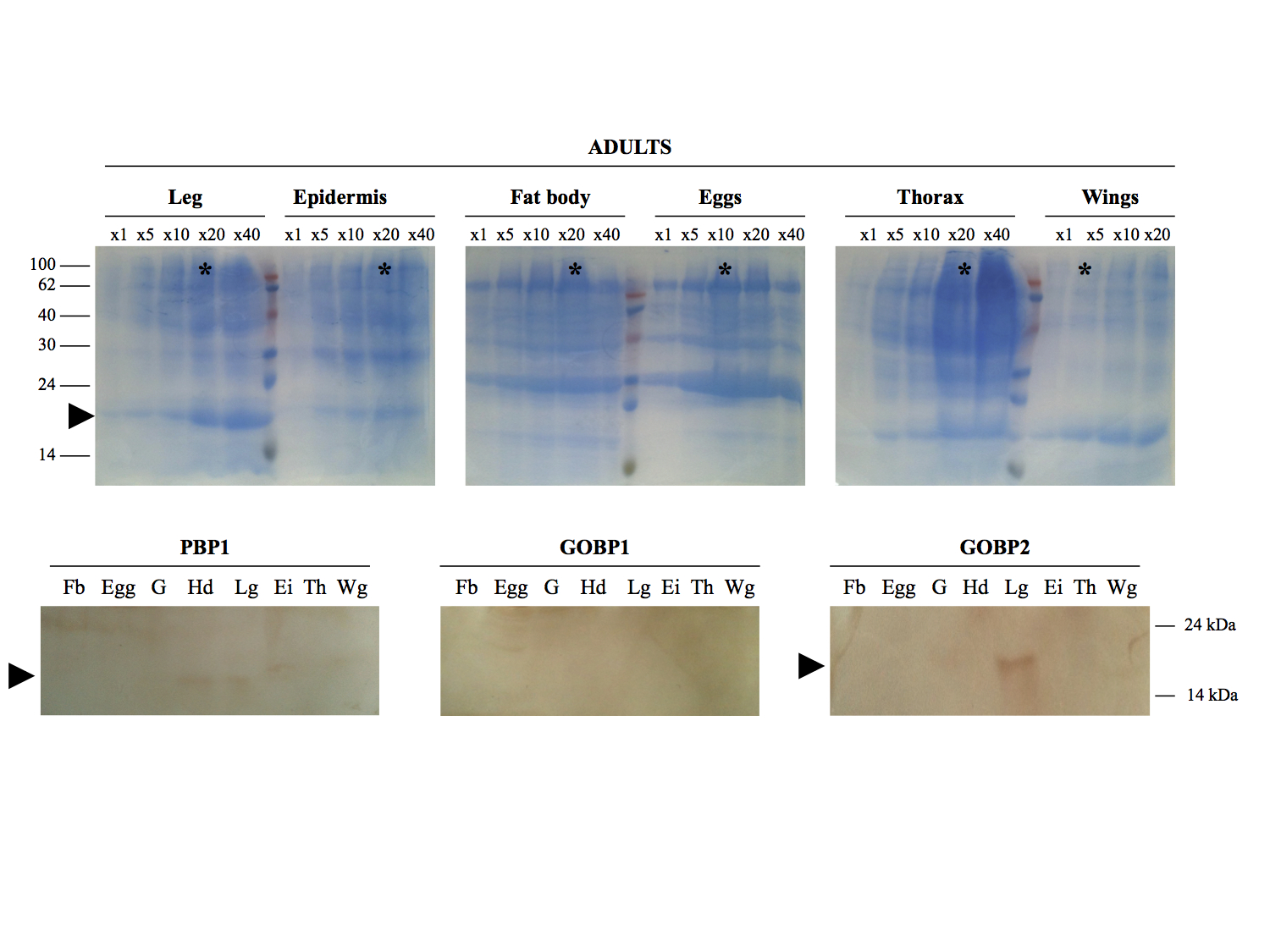

Supplement: Supplementary Figure 1 — Comparative BmorPBP1, BmorPBP2, BmorGOBP1, and BmorGOBP2 gene expression profiles across different age groups in (A) male and (B) female adult silkworm moths. Focus on OBP ratio and x-fold increase in gene expression from RNA samples (D2–D9) in the same experiment as Figure 2 (Step 1). qRT-PCR results with means (n = 9) of PBP2, GOBP1, and GOBP2 compared with PBP1 used as reference (Step 2: PBP1 expression = 1). PBP/GOBP expression aging differences between males and females. [file Data_Sheet_1.zip › Guoetal.FrontPhysiolFINAL2021-SupplMaterials/image 1.jpeg]

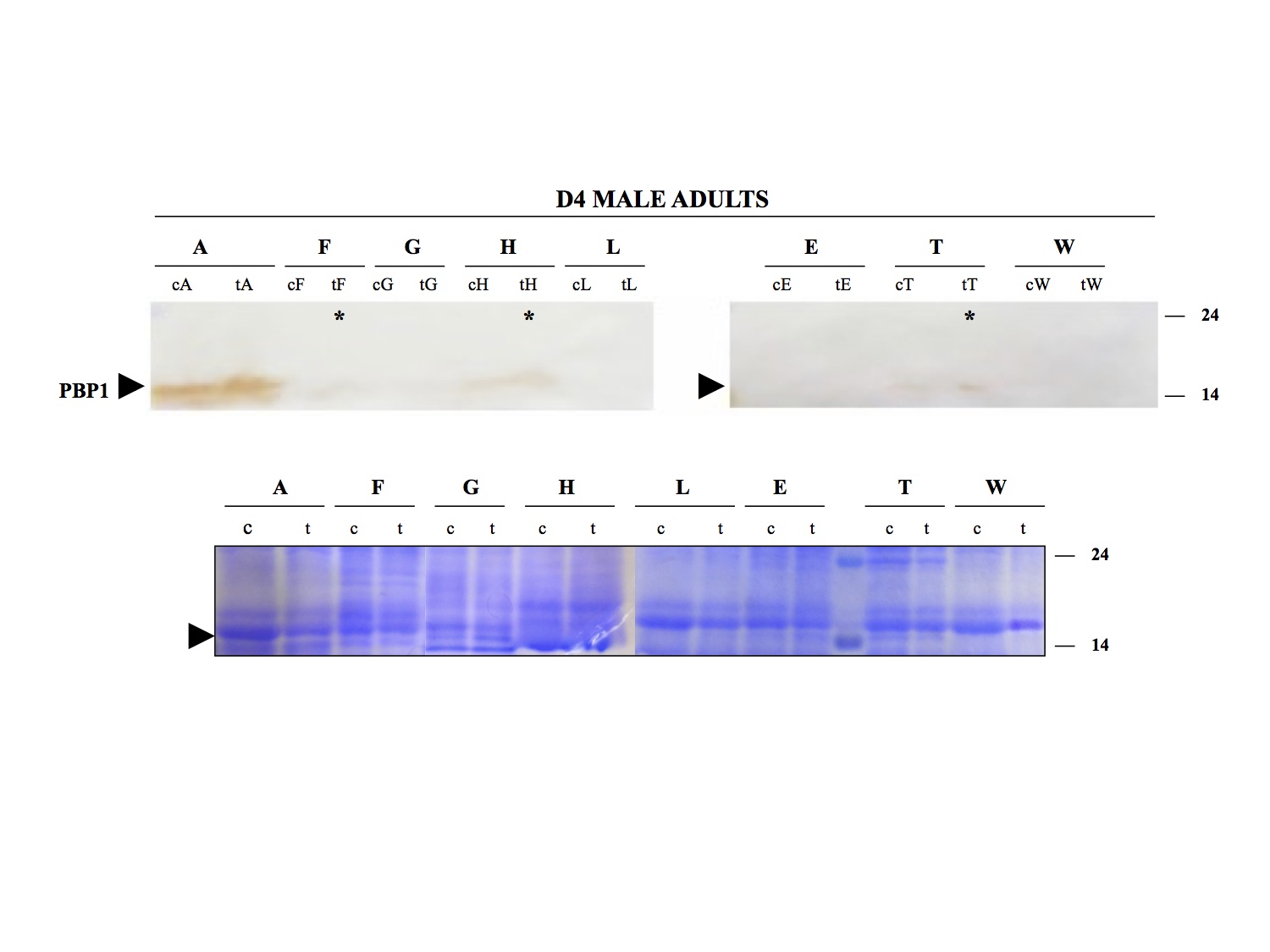

Supplement: Supplementary Figure 1 — Comparative BmorPBP1, BmorPBP2, BmorGOBP1, and BmorGOBP2 gene expression profiles across different age groups in (A) male and (B) female adult silkworm moths. Focus on OBP ratio and x-fold increase in gene expression from RNA samples (D2–D9) in the same experiment as Figure 2 (Step 1). qRT-PCR results with means (n = 9) of PBP2, GOBP1, and GOBP2 compared with PBP1 used as reference (Step 2: PBP1 expression = 1). PBP/GOBP expression aging differences between males and females. [file Data_Sheet_1.zip › Guoetal.FrontPhysiolFINAL2021-SupplMaterials/image 2.jpeg]
